# Supplementary material for: Electro-Oxidative C3-Selenylation of Pyrido[1,2-a]pyrimidin-4-ones
Source: Molecules. 2023 Feb 27;28(5):2206. doi: 10.3390/molecules28052206 (PMC10005275; doi:10.3390/molecules28052206)

Supporting Information

Electro-oxidative C3-selenylation of pyrido[1,2-*a*] pyrimidin- 4-ones

Jianwei Shi[1,†], Zhichuan Wang[2,†], Xiaoxu Teng[1], Bing Zhang[3,*], Kai Sun[2] and Xin Wang[2,*]

Table of Contents

I. General Conditions..2

II. Control Experiments..2

III. General procedure for cyclic voltammetry (CV)..................................4

IV. NMR Spectra of Products...5

I. General Conditions

All reagents were purchased from commercial sources and used without further purification. ^{1}H NMR, ^{13}C NMR spectra were recorded on a Bruker Ascend™ 400 or Bruker Ascend™ 500 spectrometer in deuterated solvents containing TMS as an internal reference standard. All high-resolution mass spectra (HRMS) were measured on a mass spectrometer by using electrospray ionization orthogonal acceleration time-of-flight (ESI-OA-TOF), and the purity of all samples used for HRMS (>95%) was confirmed by ^{1}H NMR and ^{13}C NMR spectroscopic analysis. Melting points were measured on a melting point apparatus equipped with a thermometer and were uncorrected. All the reactions were monitored by thin-layer chromatography (TLC) using GF254 silica gel-coated TLC plates. Purification by flash column chromatography was performed over SiO_2 (silica gel 200–300 mesh).

II. Control Experiments

2.1 Control experiment in the presence of TEMPO

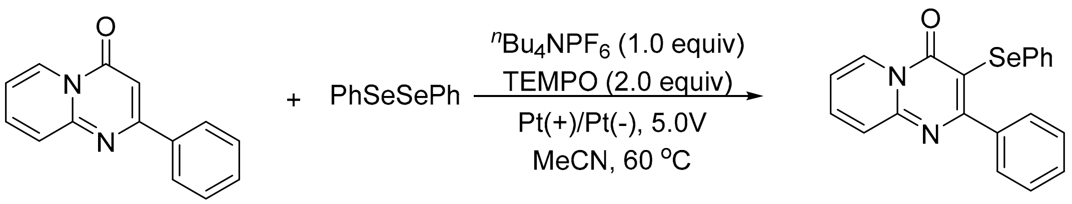

1a **2a** **3a**, 0%

2-phenyl-8,9-dihydro-4*H*-pyrido[1,2-*a*]pyrimidin-4-one **1a** (0.20 mmol, 44.42 mg), PhSeSePh **2a** (0.20 mmol, 62.43 mg), $^{n}Bu_4NPF_6$ (0.20 mmol, 77.49 mg), TEMPO (0.40 mmol, 62.50 mg) and MeCN (5.0 mL) were placed in a 10 mL two-necked round-bottomed flask. The flask was equipped with a stir bar, a platinum plate (1 cm × 1 cm) anode and a platinum plate (1 cm × 1 cm) cathode. The electrolysis was carried out under air atmosphere at 60 °C using a constant potential of 5 V for 5 h and then monitored by TLC. No desired product **3a** was detected.

2.2 Control experiment in the presence of BHT

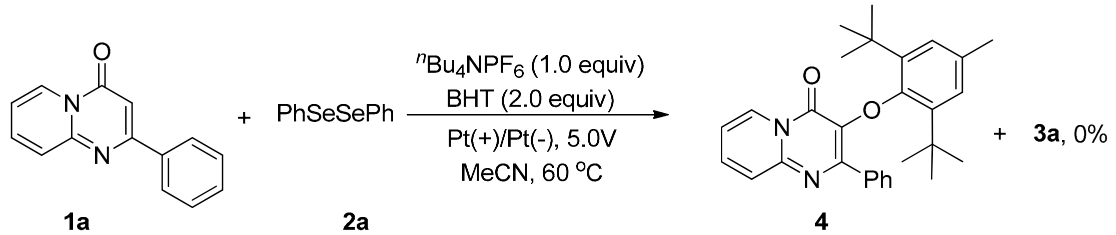

1a **2a** **4**

2-phenyl-8,9-dihydro-4*H*-pyrido[1,2-*a*]pyrimidin-4-one **1a** (0.20 mmol, 44.42 mg), PhSeSePh **2a** (0.20 mmol, 62.43 mg), $^{n}Bu_4NPF_6$ (0.20 mmol, 77.49 mg), BHT (0.40 mmol, 88.14 mg) and MeCN (5.0 mL) were placed in a 10 mL two-necked round-bottomed flask. The flask was equipped with a stir bar, a platinum plate (1 cm × 1 cm) anode and a platinum plate (1 cm × 1

cm) cathode. The electrolysis was carried out under air atmosphere at 60 °C using a constant potential of 5 V for 5 h and then monitored by TLC. The product **3a** was not obtained, and we successfully detected the desired **4** by HRMS analysis.

HRMS (ESI) calcd for $C_{29}H_{33}N_2O_2$ $[M+H]^+$: 441.2537, found: 441.2532.

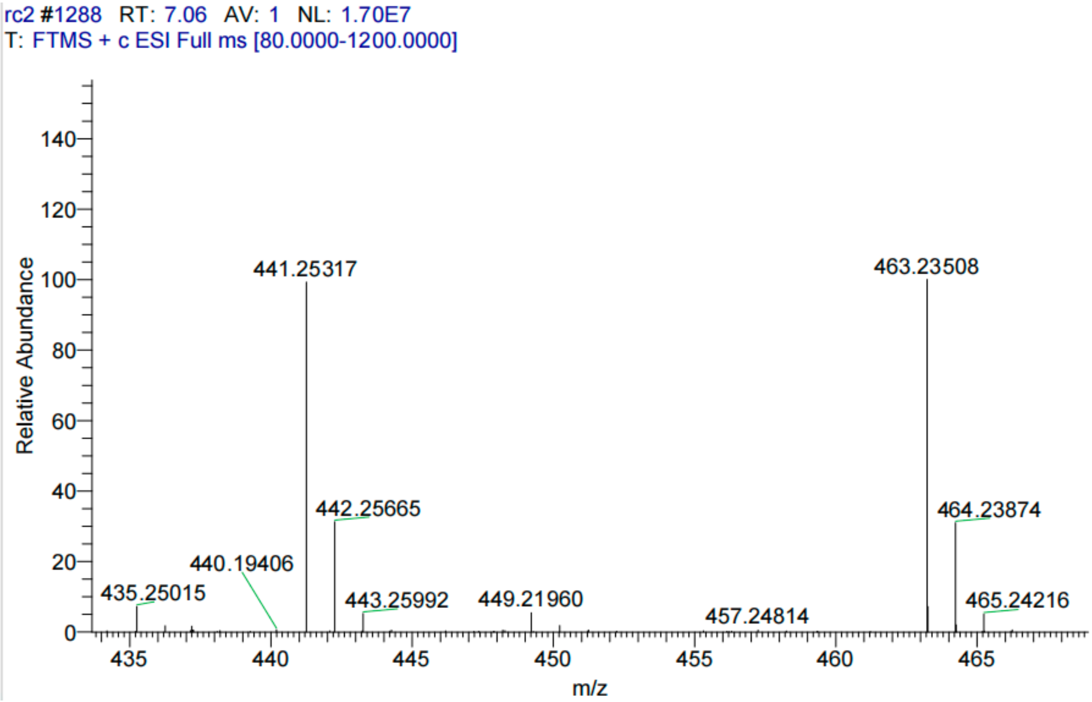

2.3 Control experiment in the presence of 1,1-Diphenylethylene

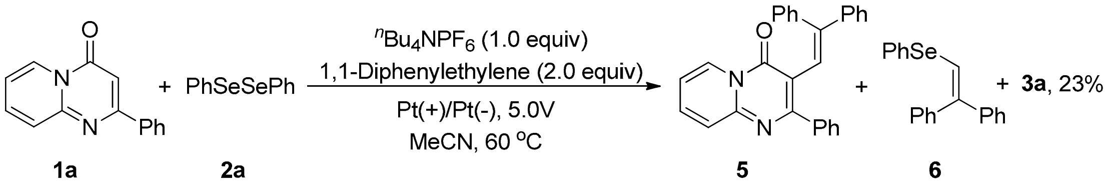

2-phenyl-8,9-dihydro-4*H*-pyrido[1,2-*a*]pyrimidin-4-one **1a** (0.20 mmol, 44.42 mg), PhSeSePh **2a** (0.20 mmol, 62.43 mg), ^{n}Bu$_4$NPF$_6$ (0.20 mmol, 77.49 mg), radical scavenger 1,1-Diphenylethylene (0.40 mmol, 108.2 mg) and MeCN (5.0 mL)were placed in a 10 mL two-necked round-bottomed flask. The flask was equipped with a stir bar, a platinum plate (1 cm × 1 cm) anode and a platinum plate (1 cm × 1 cm) cathode. The electrolysis was carried out under air atmosphere at 60 °C using a constant potential of 5 V for 5 h and then monitored by TLC. We successfully detected the desired **5** and **6** by HRMS analysis. The residue was purified by column chromatography to give the corresponding product **3a** in 23% yield.

HRMS (ESI) calcd for $C_{28}H_{21}N_2O$ $[M+H]^+$: 401.1648, found: 401.1641.

HRMS (ESI) calcd for $C_{20}H_{17}Se$ $[M+H]^+$: 337.0490, found: 337.0483.

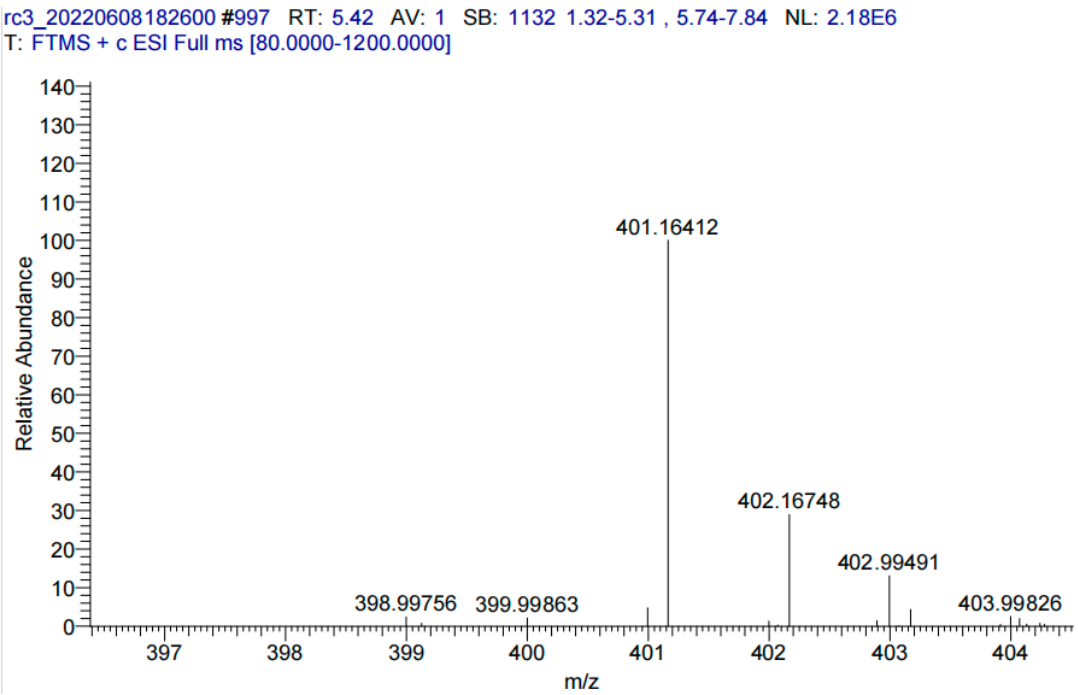

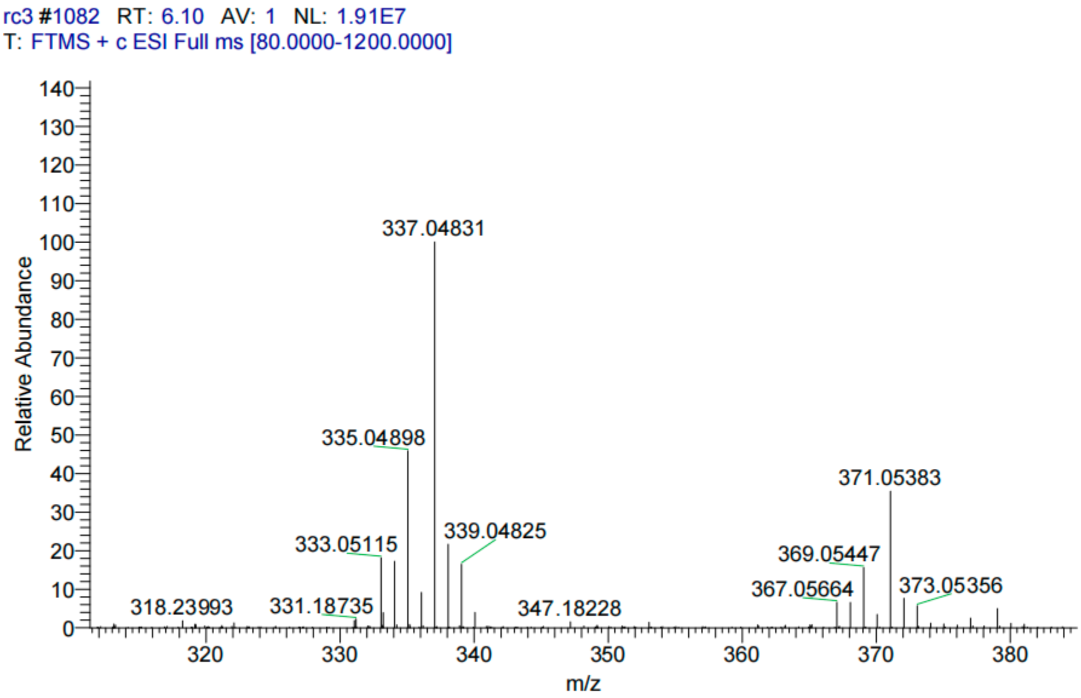

III. General procedure for cyclic voltammetry (CV)

Cyclic voltammetry was performed in a three-electrode cell connected to a Schlenk line at room temperature. The working electrode was a platinum disk electrode and the counter electrode a platinum wire. The reference was an Ag/AgCl electrode submerged in saturated aqueous KCl solution and separated from reaction by a salt bridge. Then, 10 mL of CH_3CN and 0.2 M $^{n}Bu_4NPF_6$ were poured into the electrochemical cell in all experiments. The scan rate was 0.1 V/s, ranging from 0 to 4.0 V. The peak potentials vs. Ag/AgCl were used. An obvious oxidation peak of substrate **1a** could be observed at 1.98 V (Fig. 1, blue line).

Moreover, the dihenyl diselenide **2a** demonstrated an oxidation peak at 1.88 V (Fig. 1, red line).

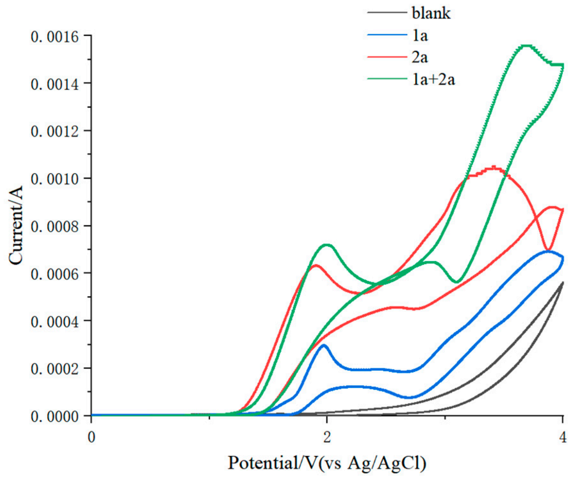

Figure 1: Cyclic voltammograms of substrate **1a**, **2a**, **1a**+**2a** (0.1 mmol) 0-4 V.

IV. NMR Spectra of Products

Compound 3a

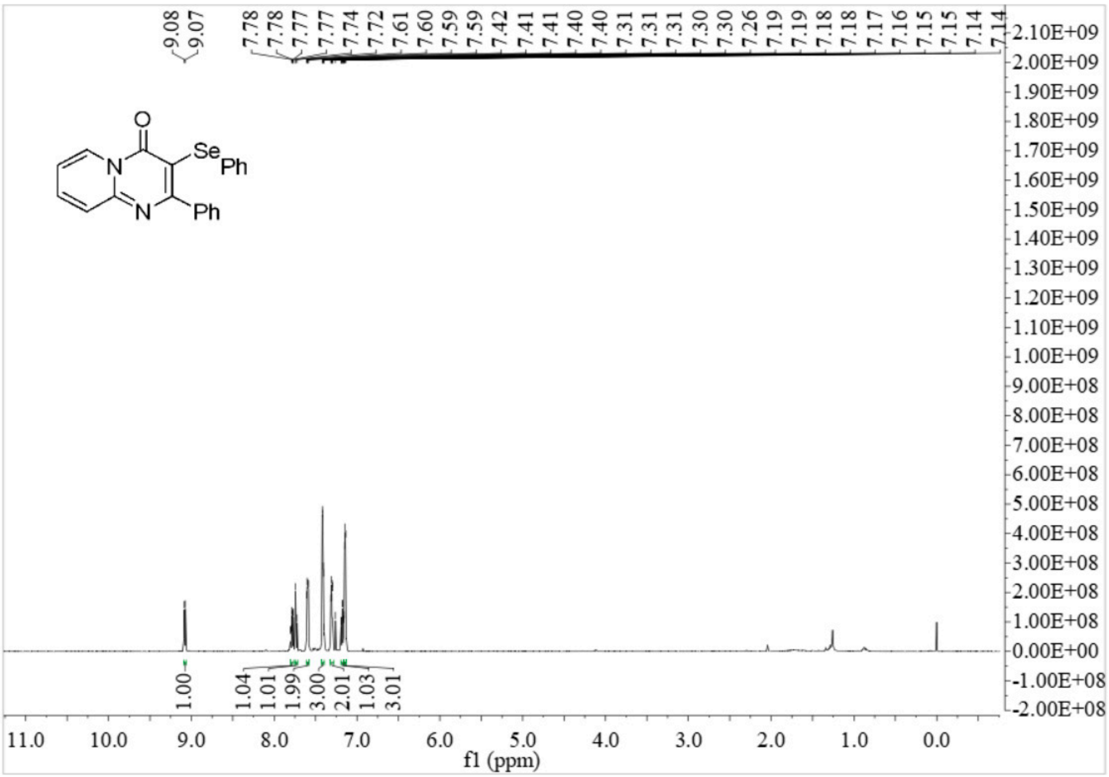

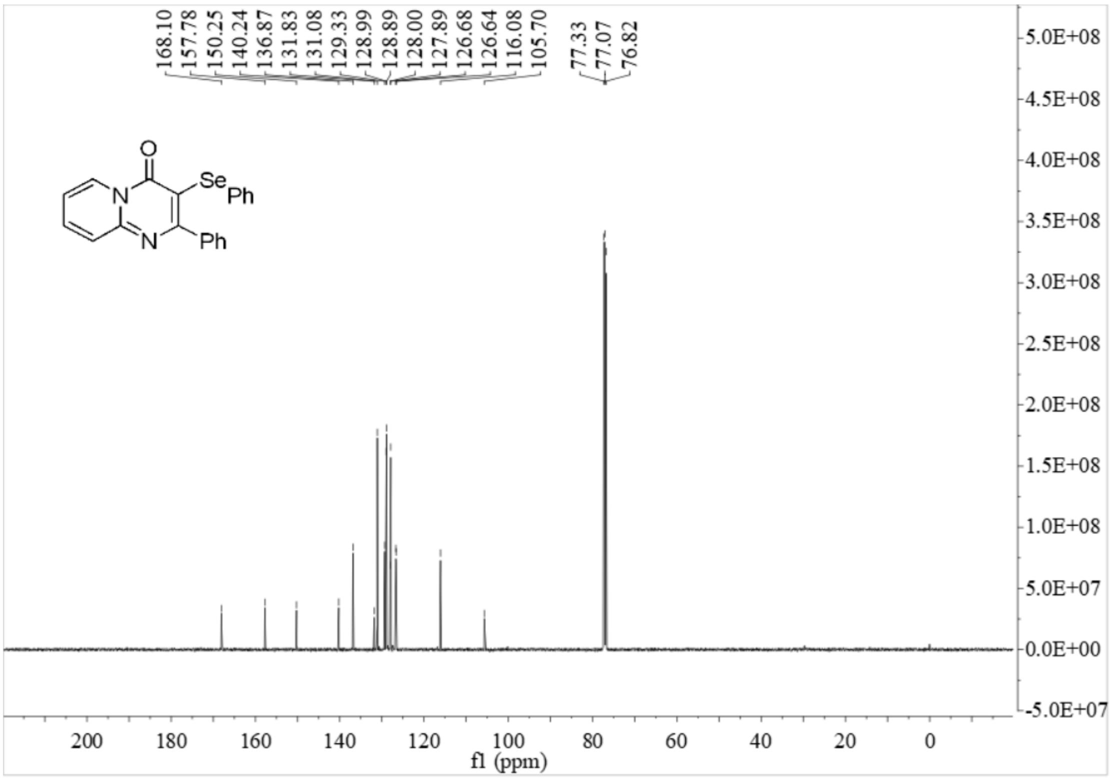
168.10
157.78
150.25
140.24
136.87
131.83
131.08
129.33
128.99
128.89
128.00
127.89
126.68
126.64
116.08
105.70
77.33
77.07
76.82
O
Se
Ph
N
N
Ph
5.0E+08
4.5E+08
4.0E+08
3.5E+08
3.0E+08
2.5E+08
2.0E+08
1.5E+08
1.0E+08
5.0E+07
0.0E+00
-5.0E+07
200
180
160
140
120
100
80
60
40
20
0
f1 (ppm)

Compound 3b

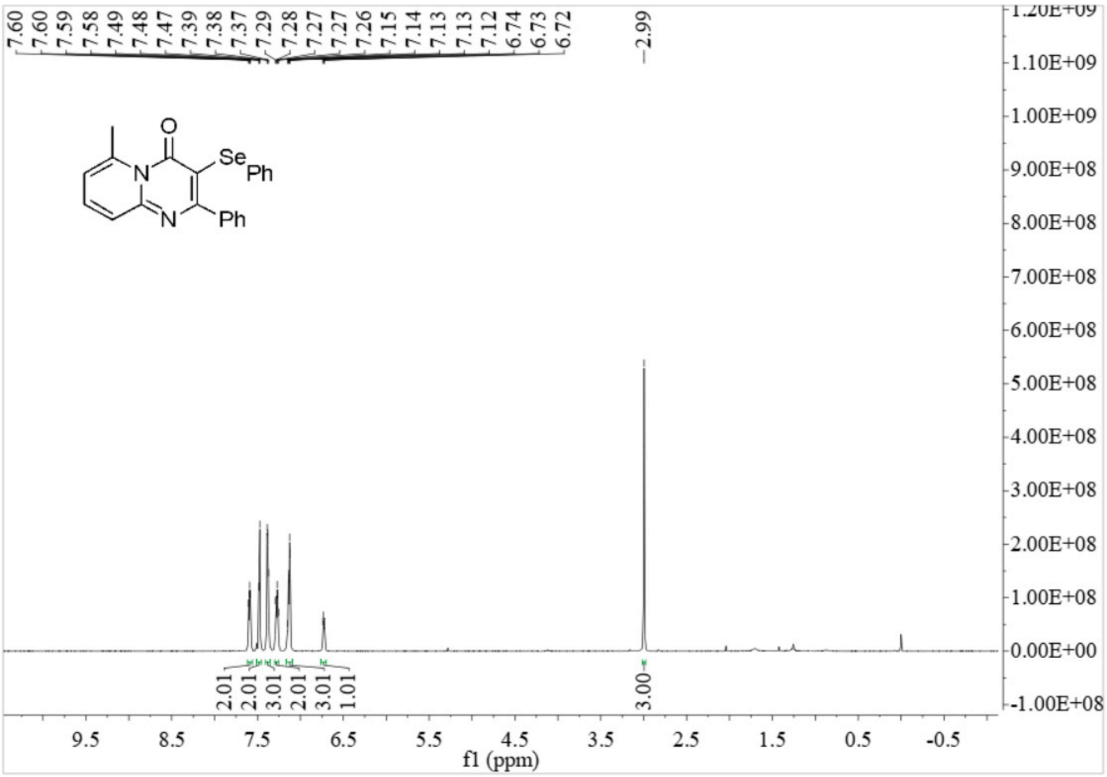
7.60
7.60
7.59
7.58
7.49
7.48
7.47
7.39
7.38
7.37
7.29
7.28
7.27
7.27
7.26
7.15
7.14
7.13
7.13
7.12
6.74
6.73
6.72
2.99
O
Se
Ph
N
N
Ph
1.20E+09
1.10E+09
1.00E+09
9.00E+08
8.00E+08
7.00E+08
6.00E+08
5.00E+08
4.00E+08
3.00E+08
2.00E+08
1.00E+08
0.00E+00
-1.00E+08
2.01
2.01
3.01
2.01
3.01
1.01
3.00
9.5
8.5
7.5
6.5
5.5
4.5
3.5
2.5
1.5
0.5
-0.5
f1 (ppm)

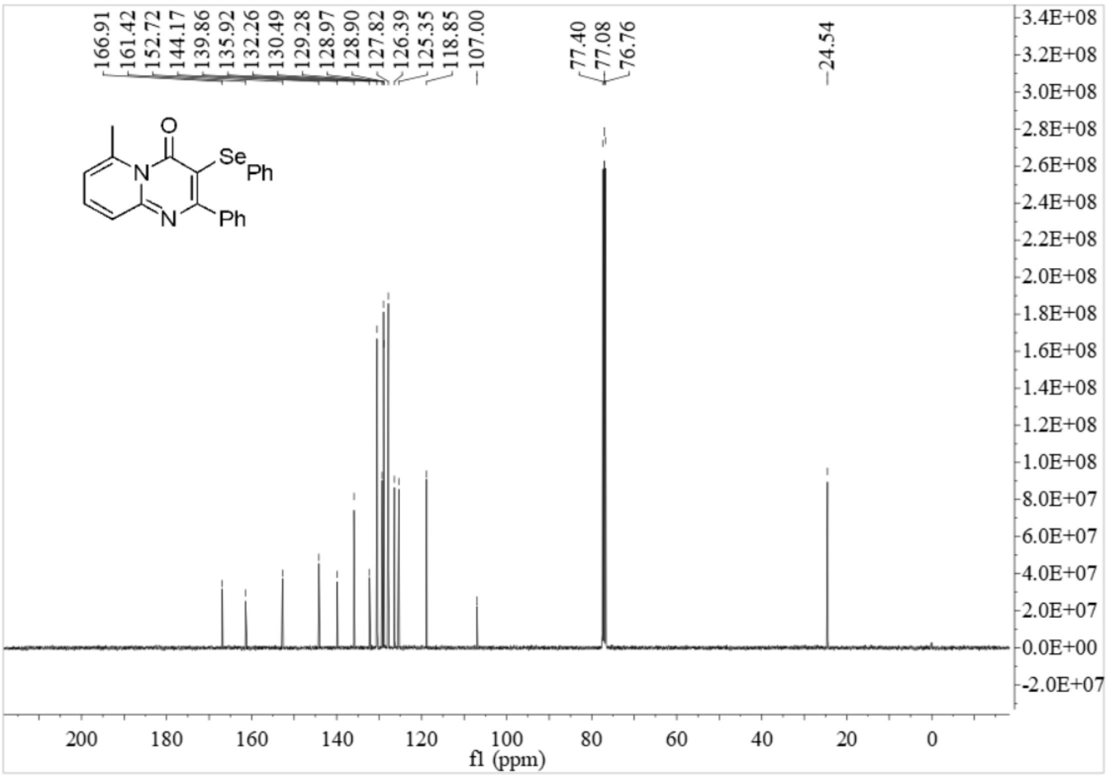
166.91
161.42
152.72
144.17
139.86
135.92
132.26
130.49
129.28
128.97
128.90
127.82
126.39
125.35
118.85
107.00
77.40
77.08
76.76
24.54
Se
Ph
O
N
N
Ph
f1 (ppm)

Compound 3c

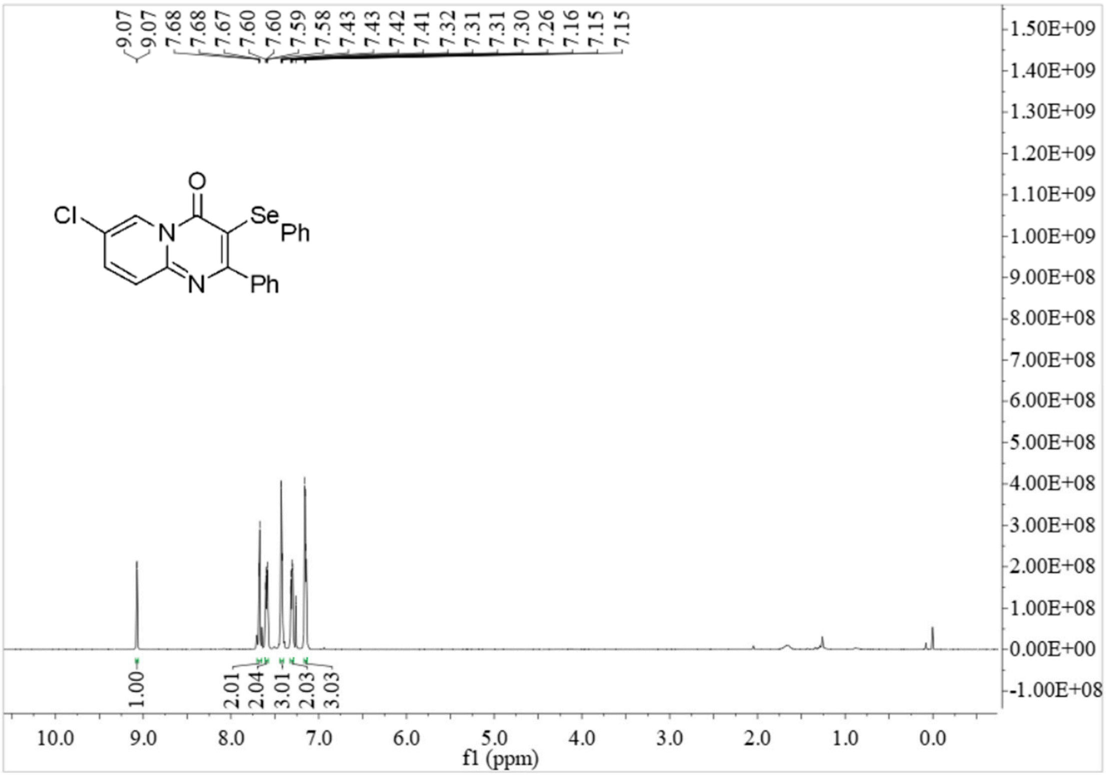
9.07
9.07
7.68
7.68
7.67
7.60
7.60
7.59
7.58
7.43
7.43
7.42
7.41
7.32
7.31
7.31
7.30
7.26
7.16
7.15
7.15
Cl
O
Se
Ph
N
N
Ph
1.00
2.01
2.04
3.01
2.03
3.03
f1 (ppm)

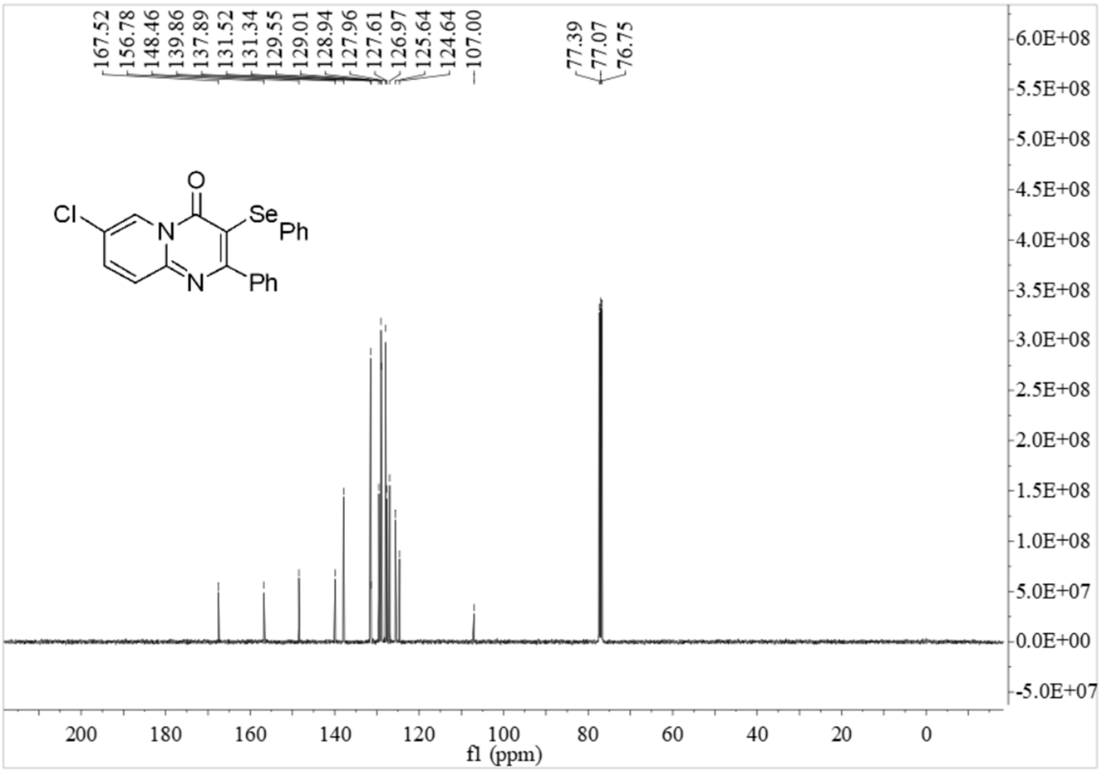
167.52
156.78
148.46
139.86
137.89
131.52
131.34
129.55
129.01
128.94
127.96
127.61
126.97
125.64
124.64
107.00
77.39
77.07
76.75
Cl
N
O
Se
Ph
N
Ph
6.0E+08
5.5E+08
5.0E+08
4.5E+08
4.0E+08
3.5E+08
3.0E+08
2.5E+08
2.0E+08
1.5E+08
1.0E+08
5.0E+07
0.0E+00
-5.0E+07
200
180
160
140
120
100
80
60
40
20
0
f1 (ppm)

Compound 3d

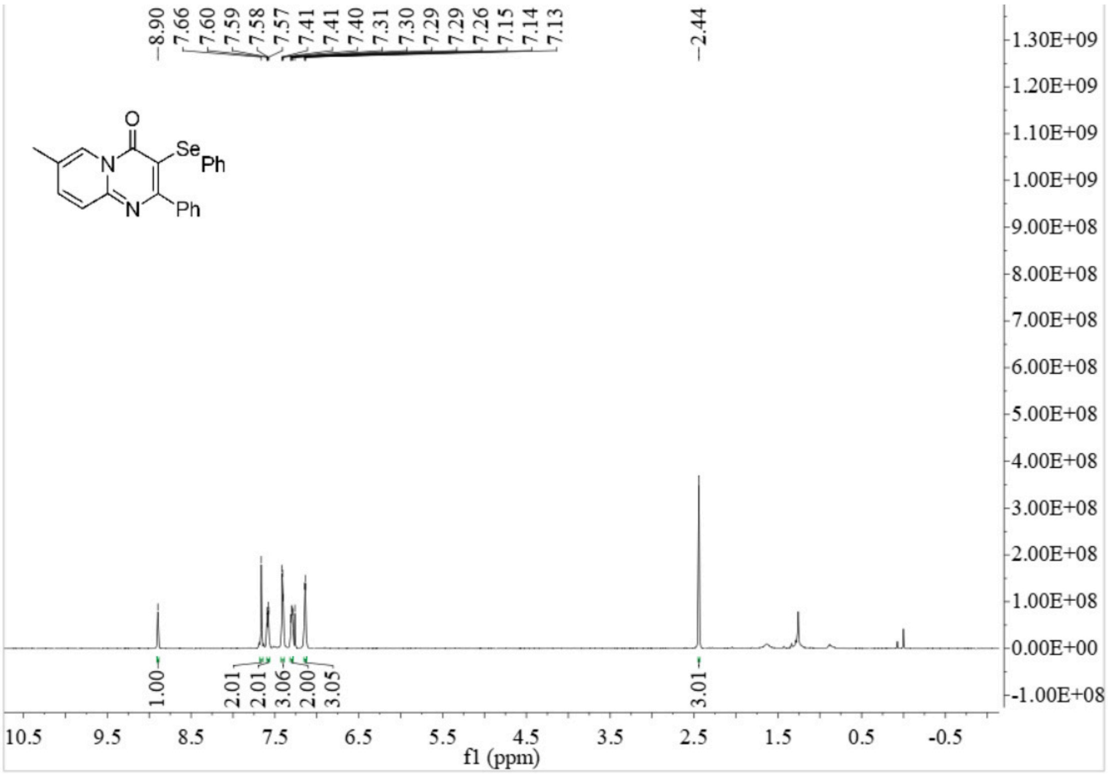
8.90
7.66
7.60
7.59
7.58
7.57
7.41
7.41
7.40
7.31
7.30
7.29
7.29
7.26
7.15
7.14
7.13
2.44
N
O
Se
Ph
N
Ph
1.30E+09
1.20E+09
1.10E+09
1.00E+09
9.00E+08
8.00E+08
7.00E+08
6.00E+08
5.00E+08
4.00E+08
3.00E+08
2.00E+08
1.00E+08
0.00E+00
-1.00E+08
1.00
2.01
2.01
3.06
2.00
3.05
3.01
10.5
9.5
8.5
7.5
6.5
5.5
4.5
3.5
2.5
1.5
0.5
-0.5
f1 (ppm)

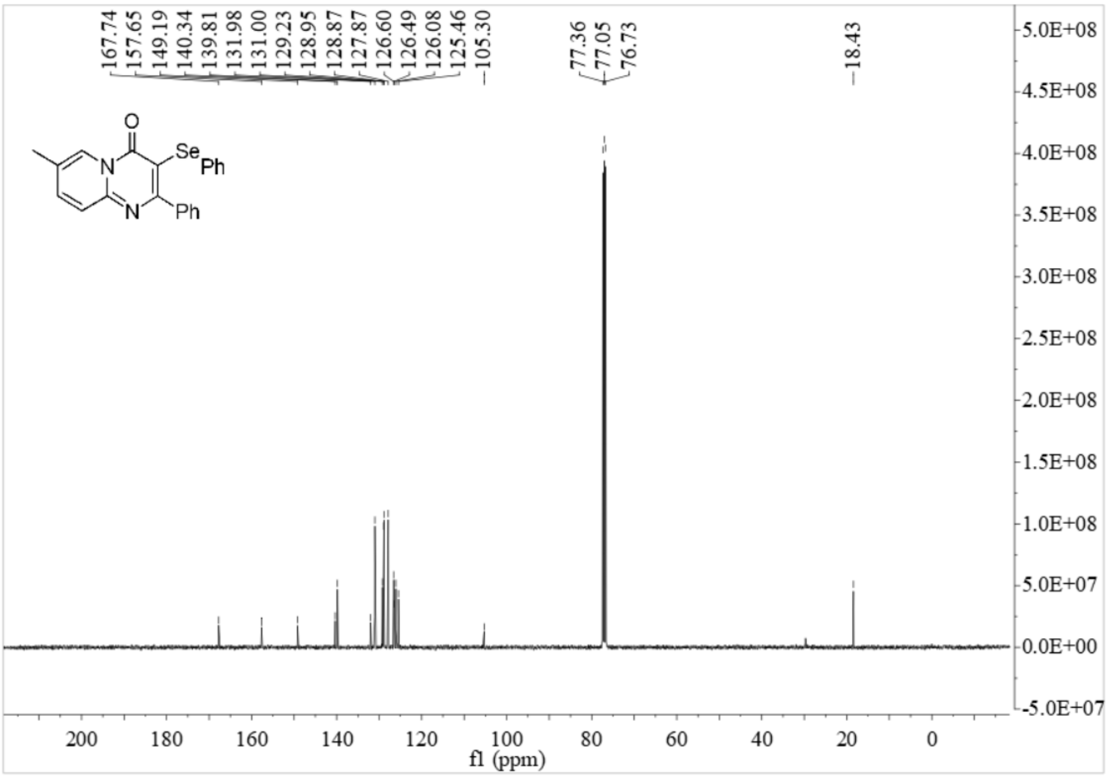

167.74
157.65
149.19
140.34
139.81
131.98
131.00
129.23
128.95
128.87
127.87
126.60
126.49
126.08
125.46
105.30
77.36
77.05
76.73
18.43
Se
Ph
N
O
N
Ph
5.0E+08
4.5E+08
4.0E+08
3.5E+08
3.0E+08
2.5E+08
2.0E+08
1.5E+08
1.0E+08
5.0E+07
0.0E+00
-5.0E+07
200
180
160
140
120
100
80
60
40
20
0
f1 (ppm)

Compound 3e

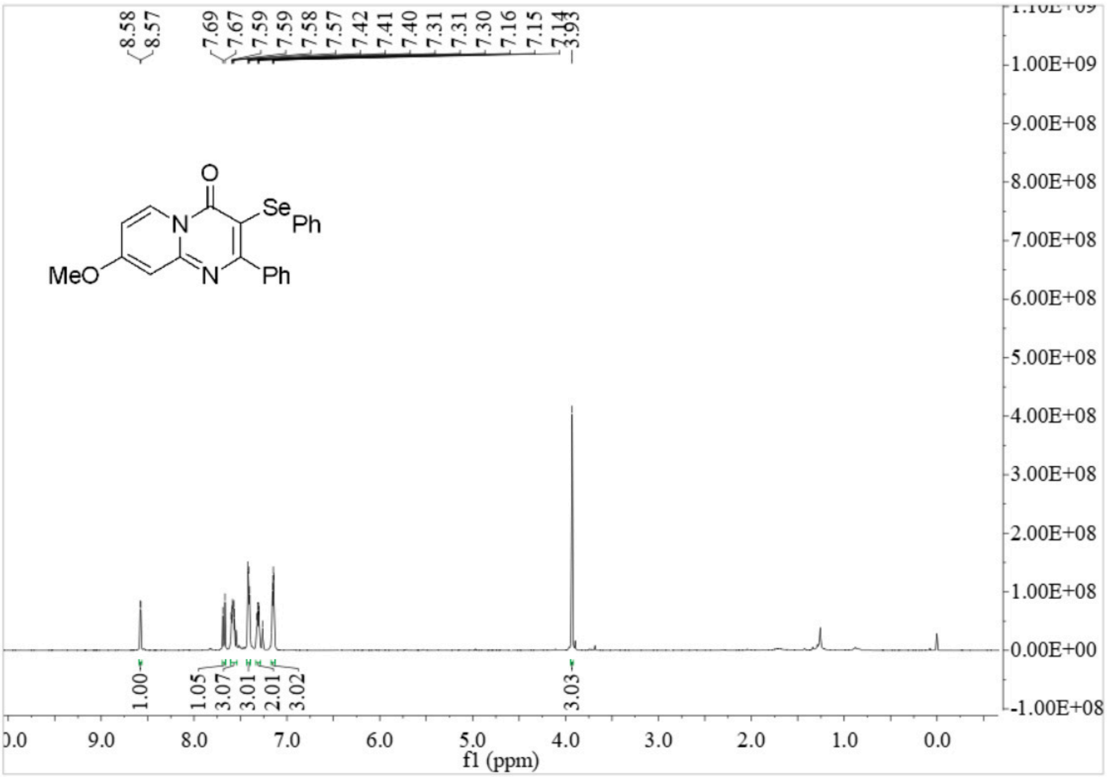
8.58
8.57
7.69
7.67
7.59
7.59
7.58
7.57
7.42
7.41
7.40
7.31
7.31
7.30
7.16
7.15
7.14
3.93
O
Se
Ph
N
N
MeO
Ph
1.00
1.05
3.07
3.01
2.01
3.02
3.03
f1 (ppm)

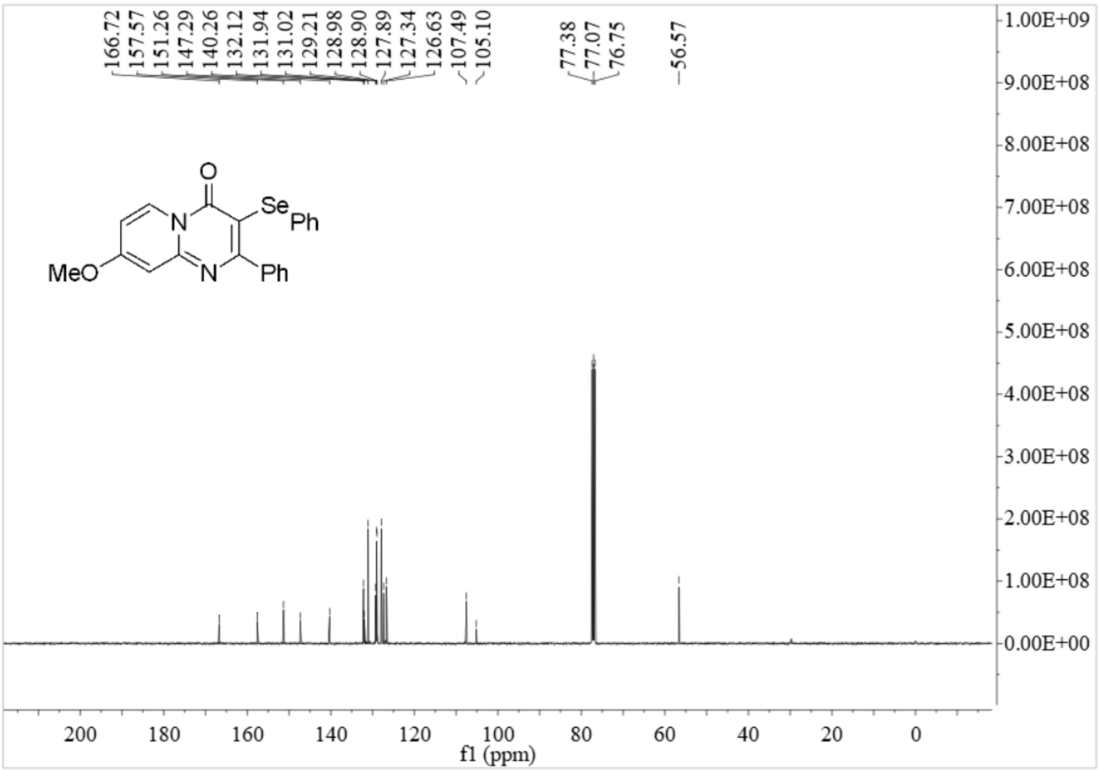
166.72
157.57
151.26
147.29
140.26
132.12
131.94
131.02
129.21
128.98
128.90
127.89
127.34
126.63
107.49
105.10
77.38
77.07
76.75
56.57
O
Se
Ph
N
N
MeO
Ph
f1 (ppm)

Compound 3f

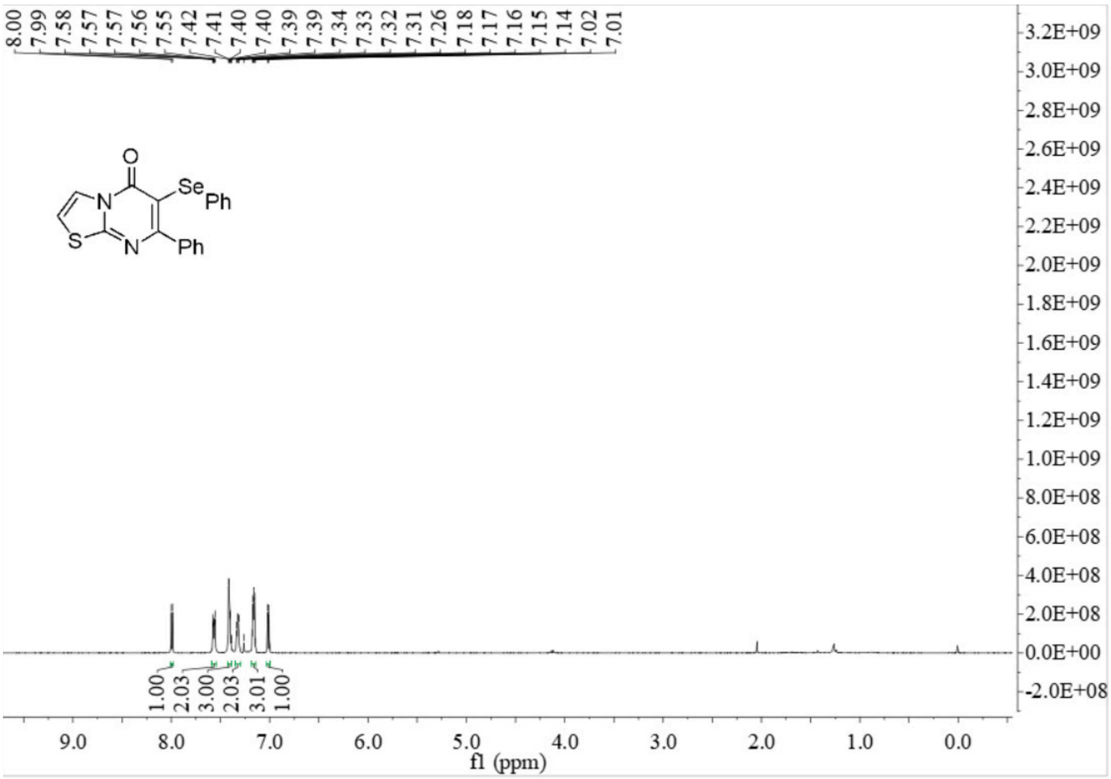
8.00
7.99
7.58
7.57
7.57
7.56
7.55
7.42
7.41
7.40
7.40
7.39
7.39
7.34
7.33
7.32
7.31
7.26
7.18
7.17
7.16
7.15
7.14
7.02
7.01
O
Se
Ph
N
S
N
Ph
1.00
2.03
3.00
2.03
3.01
1.00
f1 (ppm)

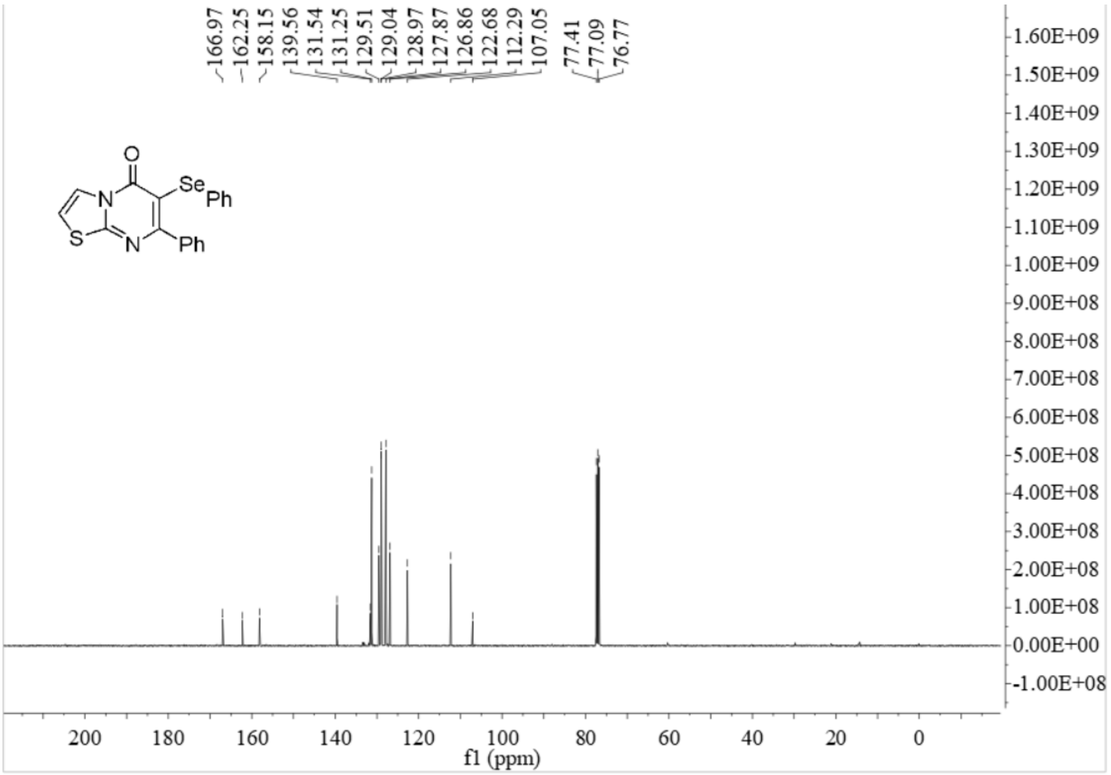
166.97
162.25
158.15
139.56
131.54
131.25
129.51
129.04
128.97
127.87
126.86
122.68
112.29
107.05
77.41
77.09
76.77
O
Se
Ph
N
S
N
Ph
f1 (ppm)

Compound 3g

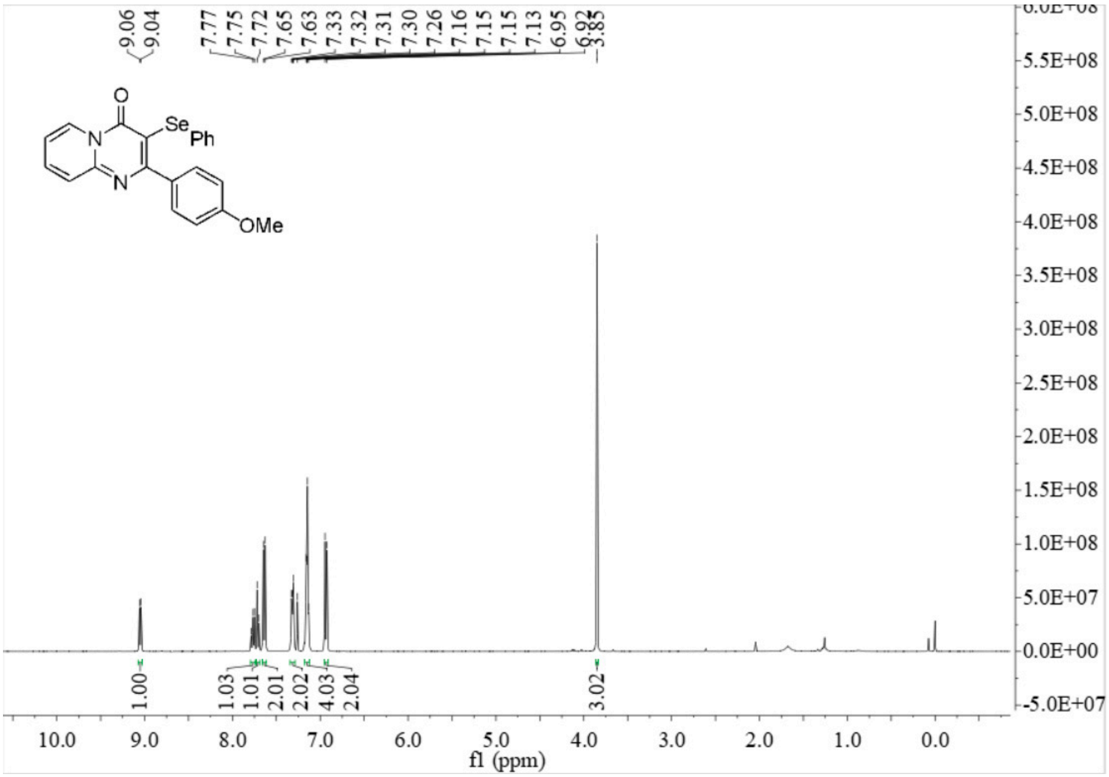
9.06
9.04
7.77
7.75
7.72
7.65
7.63
7.33
7.32
7.31
7.30
7.26
7.16
7.15
7.15
7.13
6.95
6.92
3.85
Se
Ph
N
N
O
OMe
1.00
1.03
1.01
2.01
2.02
4.03
2.04
3.02
10.0
9.0
8.0
7.0
6.0
5.0
4.0
3.0
2.0
1.0
0.0
f1 (ppm)
5.5E+08
5.0E+08
4.5E+08
4.0E+08
3.5E+08
3.0E+08
2.5E+08
2.0E+08
1.5E+08
1.0E+08
5.0E+07
0.0E+00
-5.0E+07

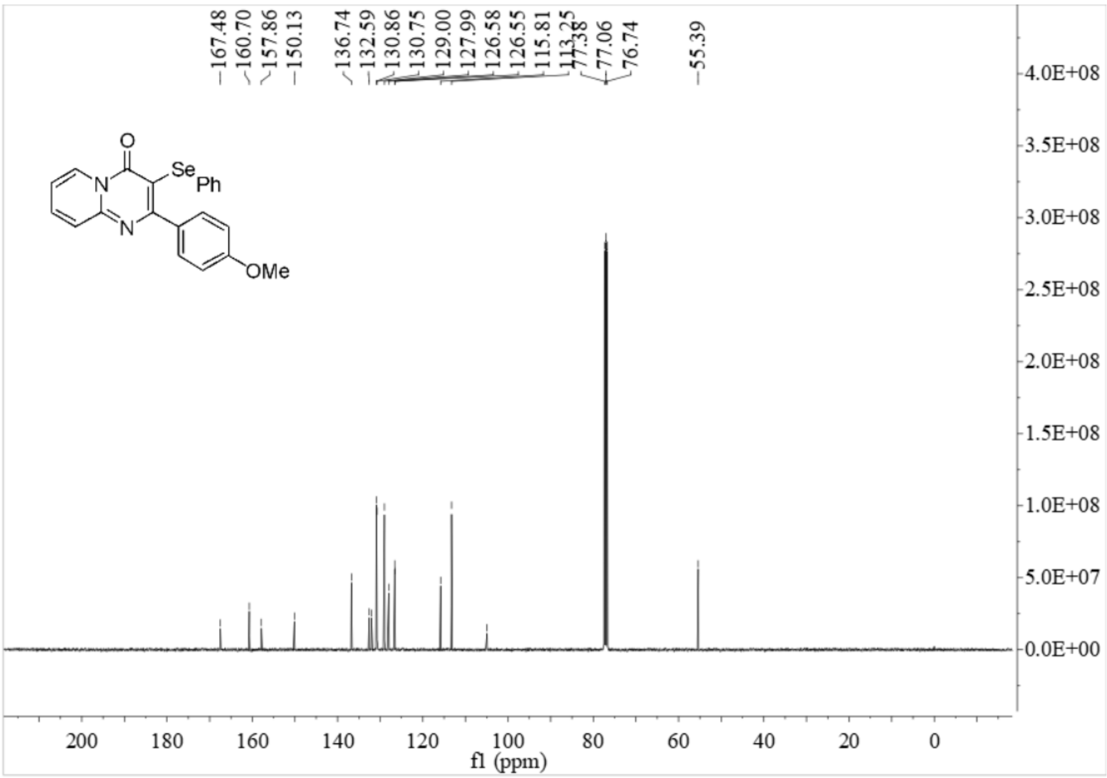
167.48
160.70
157.86
150.13
136.74
132.59
130.86
130.75
129.00
127.99
126.58
126.55
115.81
113.25
77.38
77.06
76.74
55.39
Se
Ph
N
N
O
OMe
200
180
160
140
120
100
80
60
40
20
0
f1 (ppm)
4.0E+08
3.5E+08
3.0E+08
2.5E+08
2.0E+08
1.5E+08
1.0E+08
5.0E+07
0.0E+00

Compound 3h

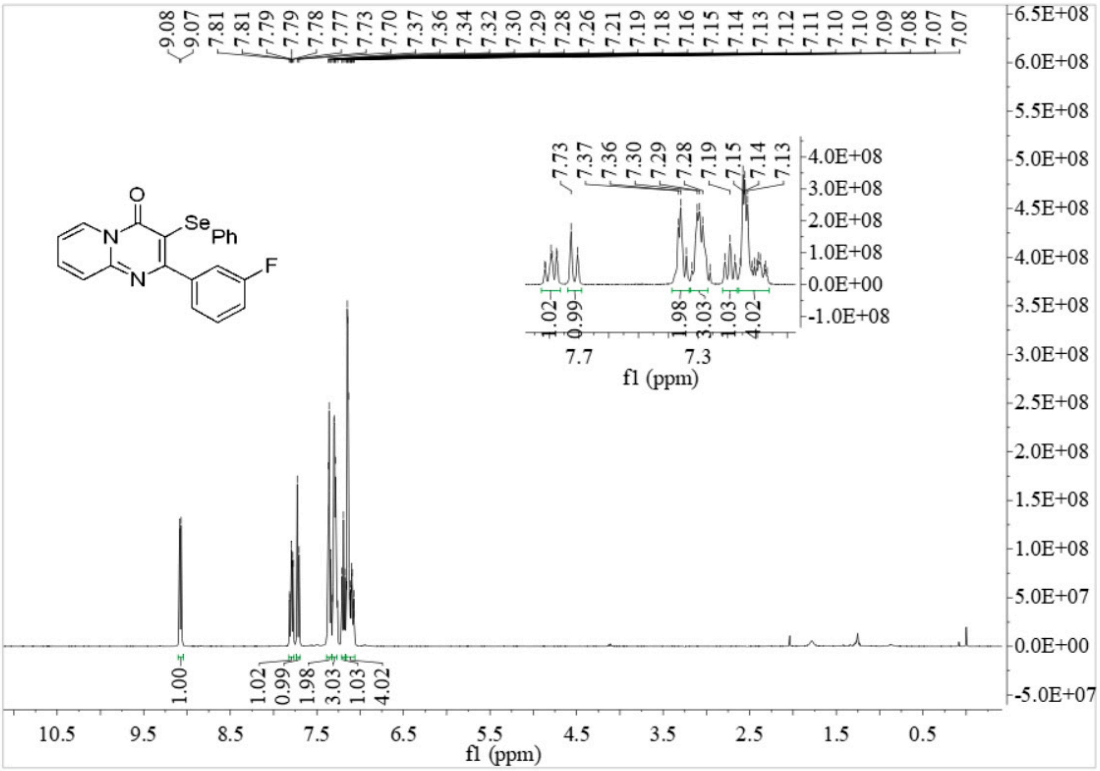
9.08
9.07
7.81
7.81
7.79
7.79
7.78
7.77
7.73
7.70
7.37
7.36
7.34
7.32
7.30
7.29
7.28
7.26
7.21
7.19
7.18
7.16
7.15
7.14
7.13
7.12
7.11
7.10
7.10
7.09
7.08
7.07
7.07
Se
Ph
O
N
N
F
1.00
1.02
0.99
1.98
3.03
1.03
4.02
f1 (ppm)

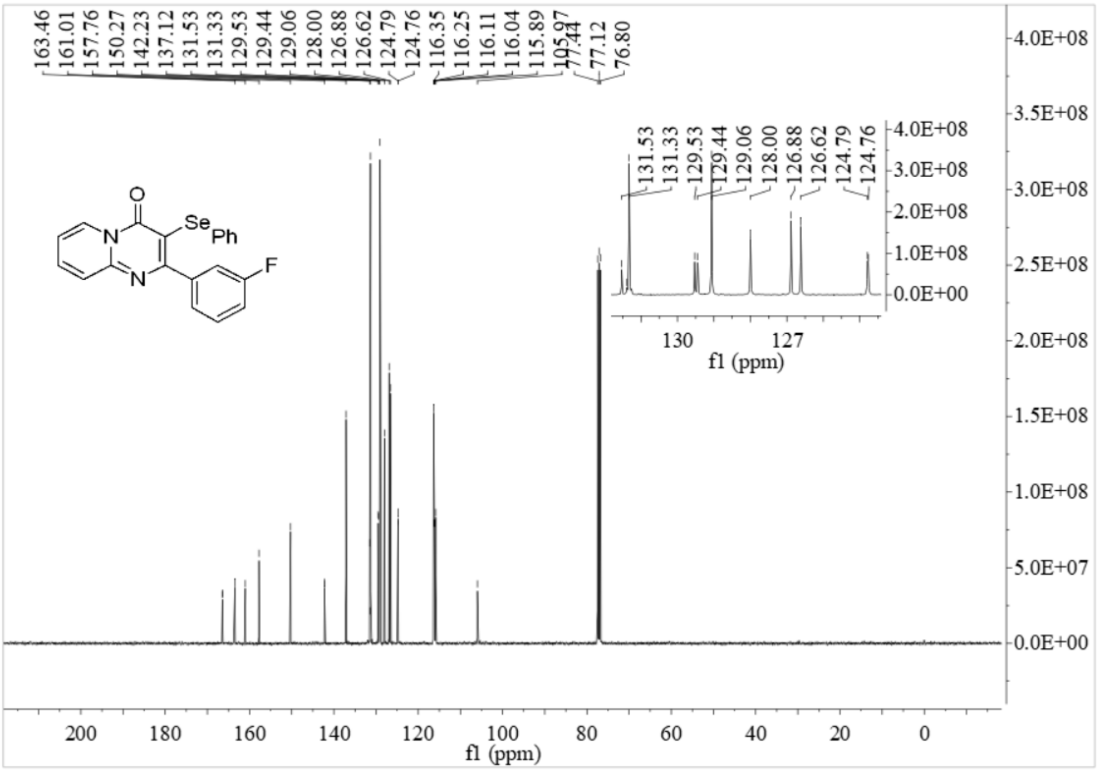
163.46
161.01
157.76
150.27
142.23
137.12
131.53
131.33
129.53
129.44
129.06
128.00
126.88
126.62
124.79
124.76
116.35
116.25
116.11
116.04
115.89
105.97
77.44
77.12
76.80
Se
Ph
O
N
N
F
f1 (ppm)

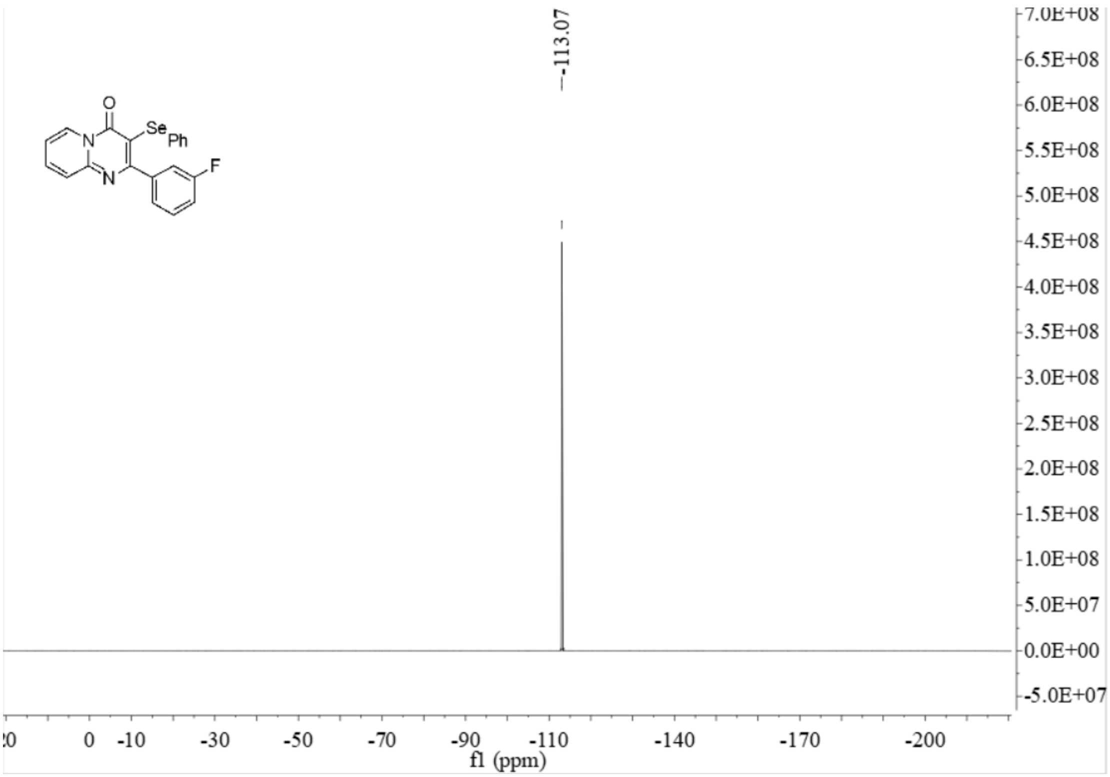

-113.07
Se
Ph
N
N
O
F
7.0E+08
6.5E+08
6.0E+08
5.5E+08
5.0E+08
4.5E+08
4.0E+08
3.5E+08
3.0E+08
2.5E+08
2.0E+08
1.5E+08
1.0E+08
5.0E+07
0.0E+00
-5.0E+07
0
-10
-30
-50
-70
-90
-110
-140
-170
-200
f1 (ppm)

Compound 3i

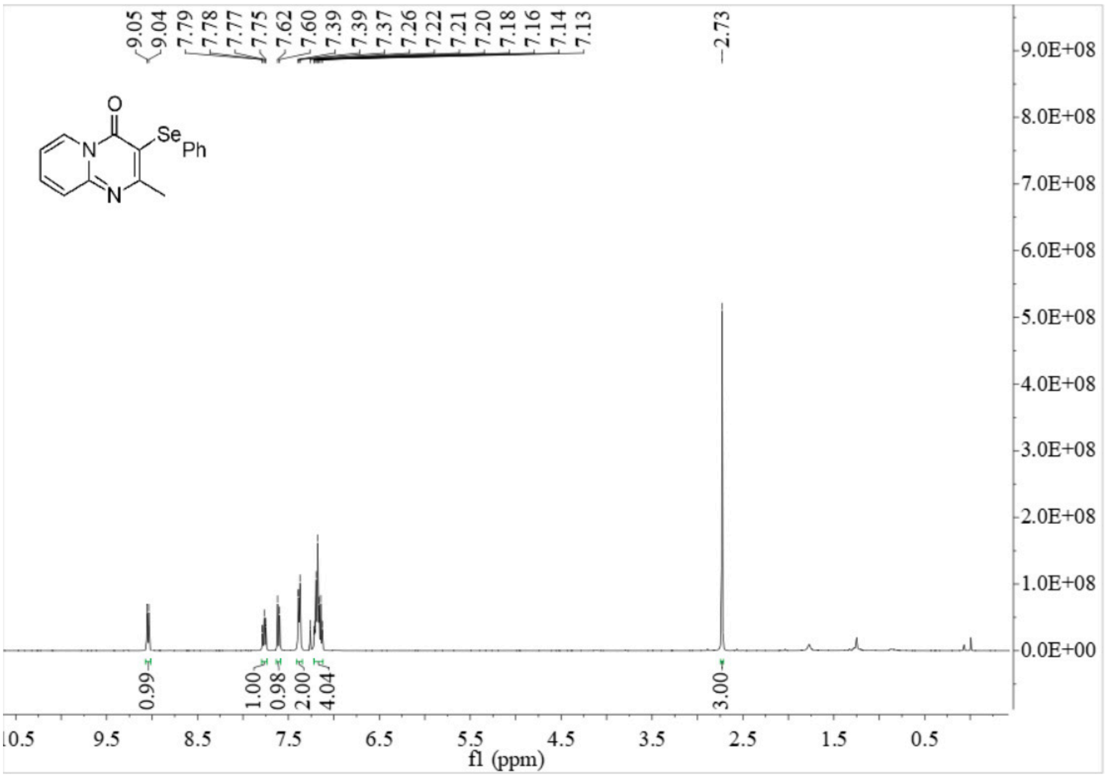
9.05
9.04
7.79
7.78
7.77
7.75
7.62
7.60
7.39
7.39
7.37
7.26
7.22
7.21
7.20
7.18
7.16
7.14
7.13
2.73
Se
Ph
O
N
N
0.99
1.00
0.98
2.00
4.04
3.00
f1 (ppm)

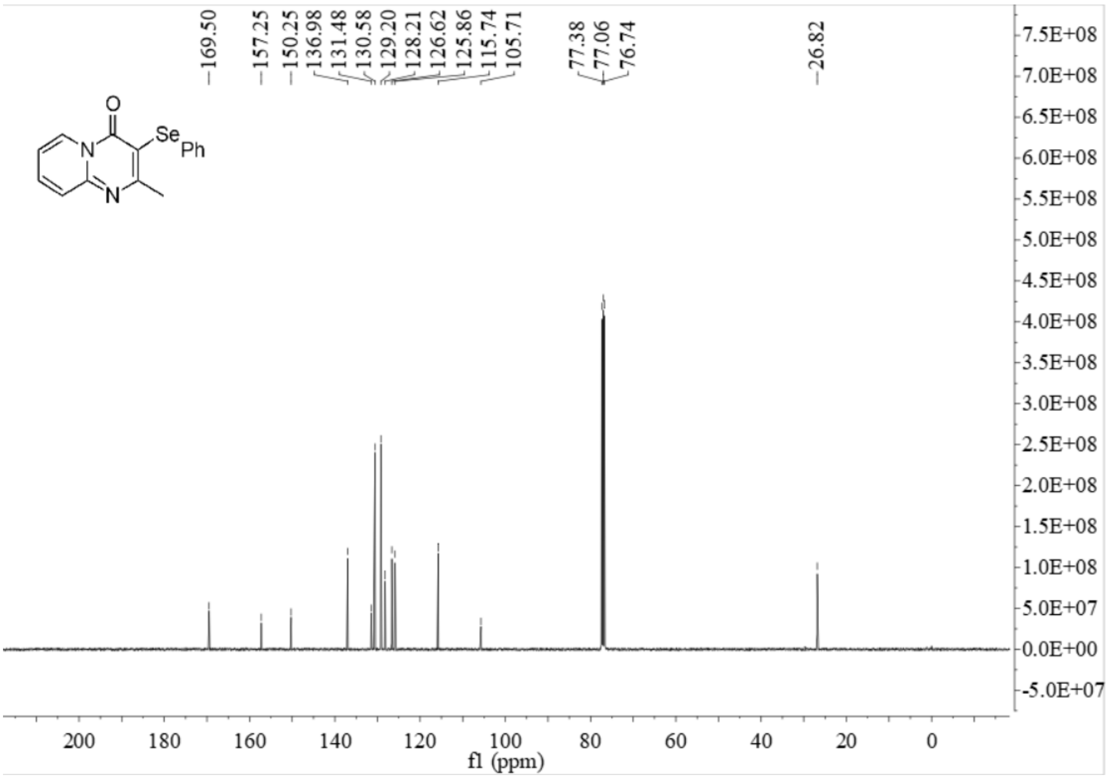
169.50
157.25
150.25
136.98
131.48
130.58
129.20
128.21
126.62
125.86
115.74
105.71
77.38
77.06
76.74
26.82
f1 (ppm)

Compound 3j

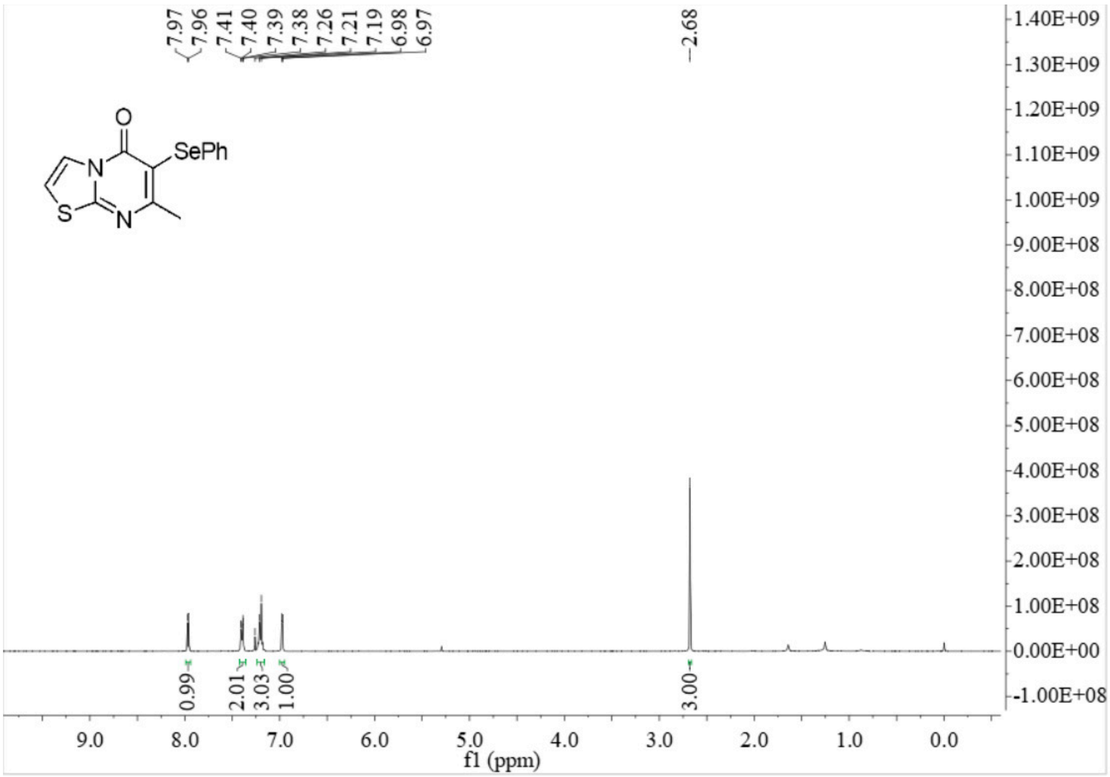
7.97
7.96
7.41
7.40
7.39
7.38
7.26
7.21
7.19
6.98
6.97
2.68
SePh
S
N
0.99
2.01
3.03
1.00
3.00
f1 (ppm)

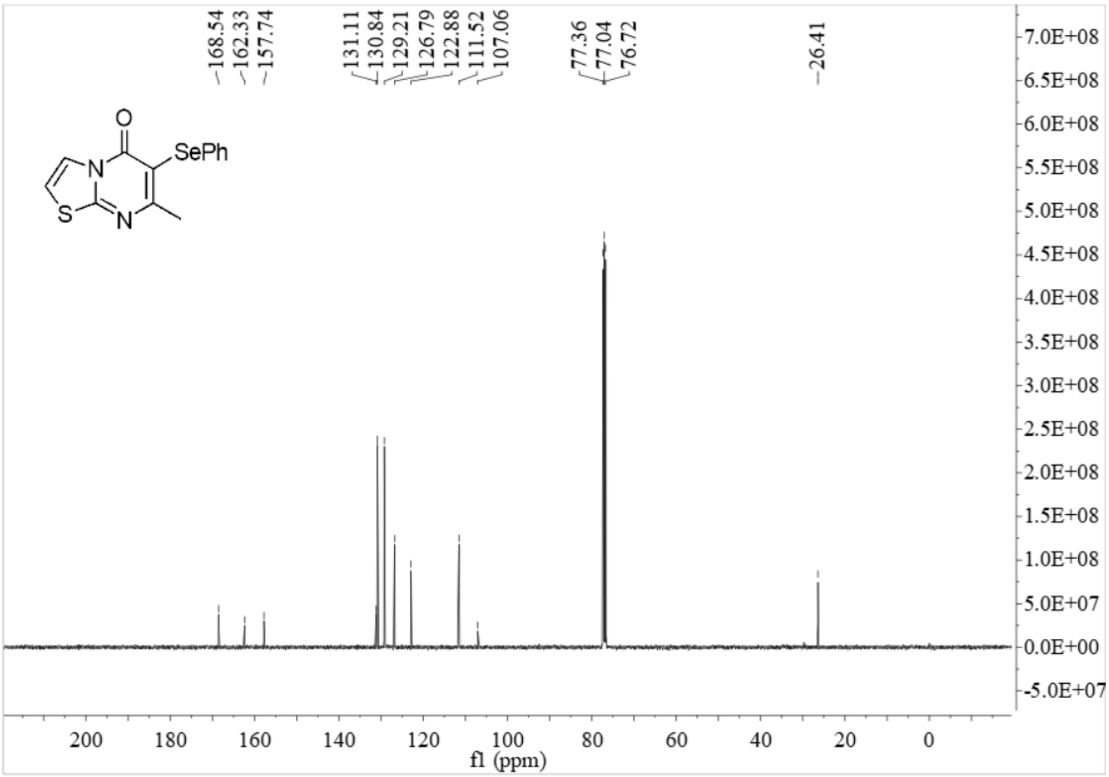
168.54
162.33
157.74
131.11
130.84
129.21
126.79
122.88
111.52
107.06
77.36
77.04
76.72
26.41
SePh
S
N
f1 (ppm)

Compound 3k

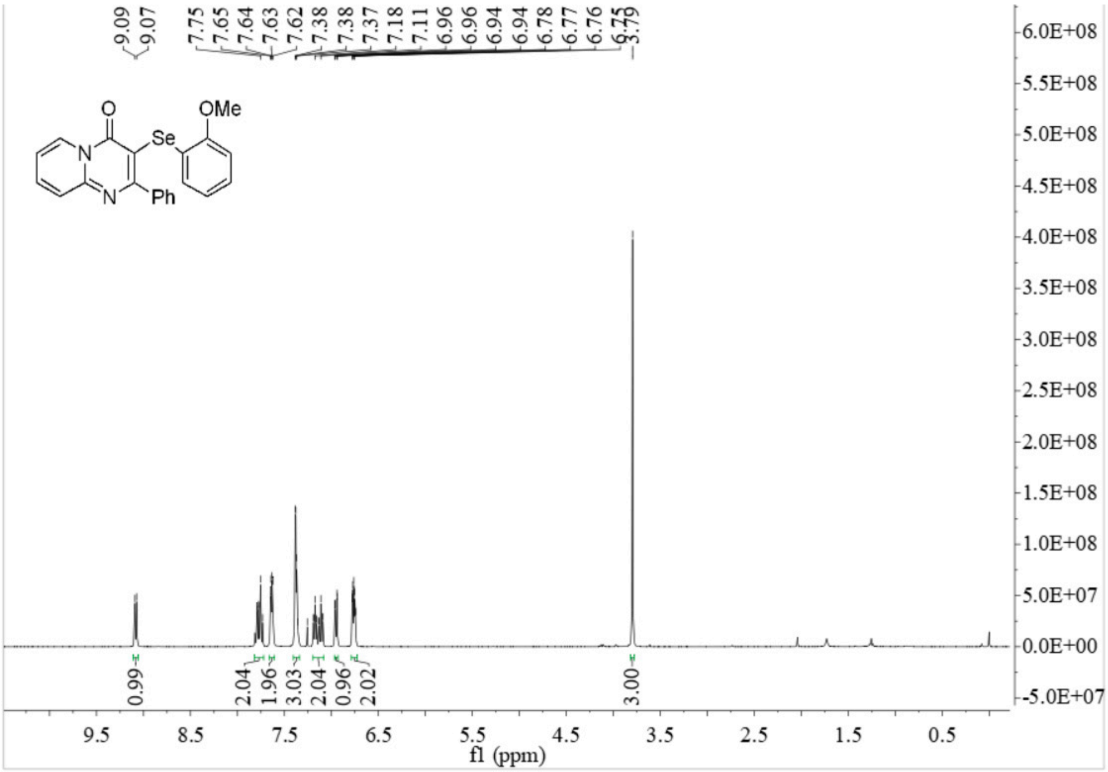

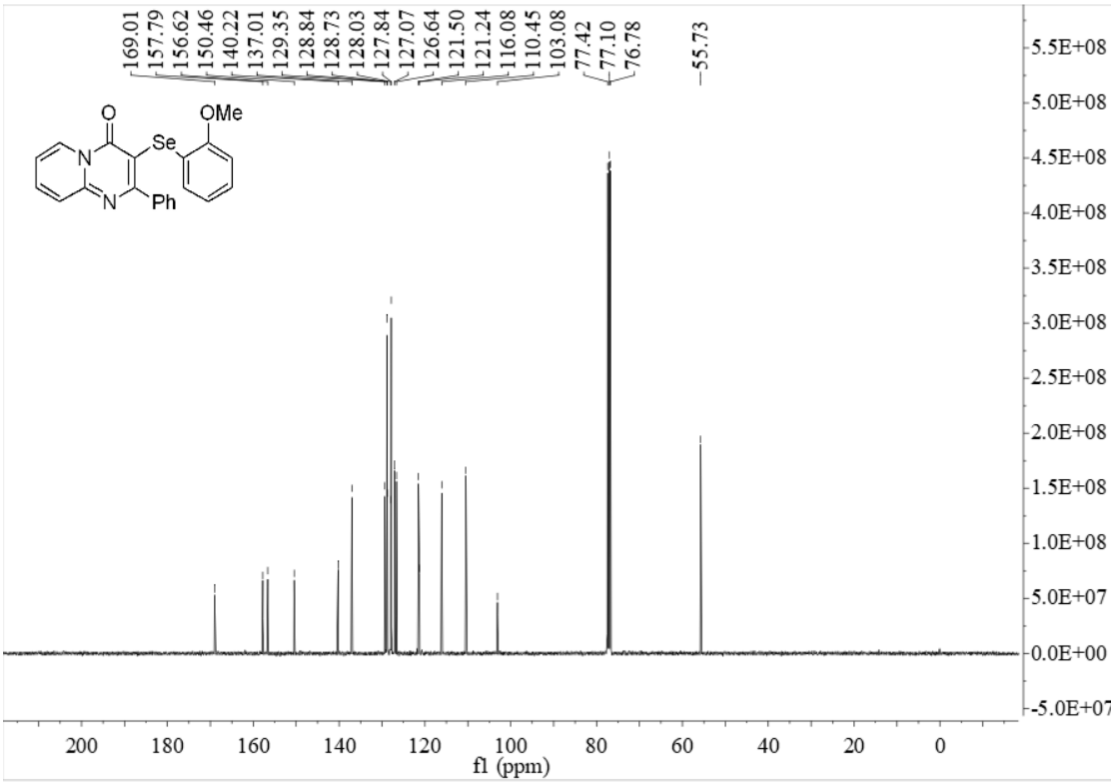

Compound 3l

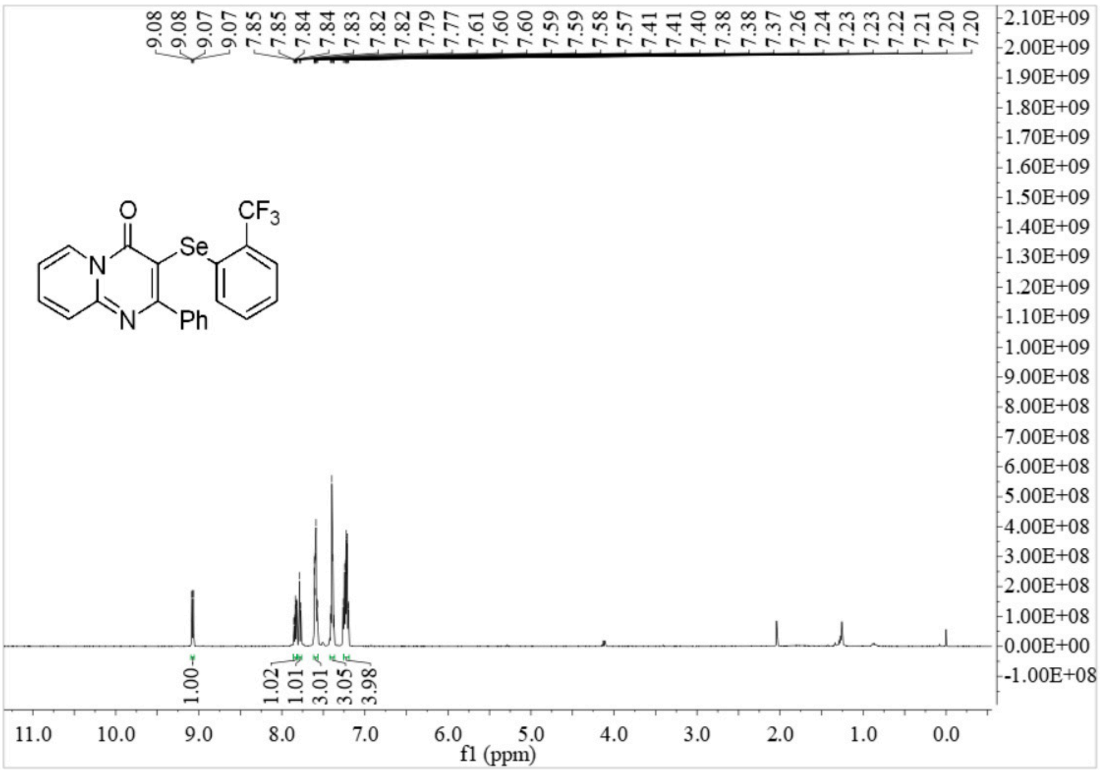
9.08
9.08
9.07
9.07
7.85
7.85
7.84
7.84
7.83
7.82
7.82
7.79
7.77
7.61
7.60
7.60
7.59
7.59
7.58
7.57
7.41
7.41
7.40
7.38
7.38
7.37
7.26
7.24
7.23
7.23
7.22
7.21
7.20
7.20
1.00
1.02
1.01
3.01
3.05
3.98
f1 (ppm)

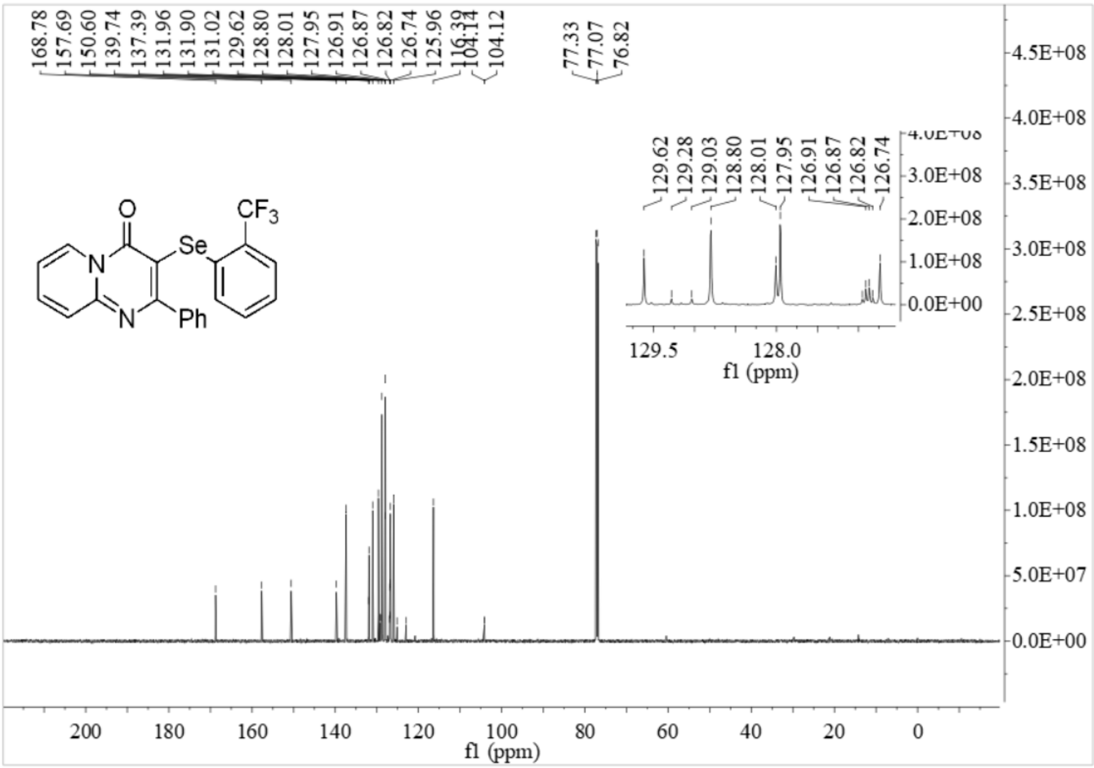
168.78
157.69
150.60
139.74
137.39
131.96
131.90
131.02
129.62
128.80
128.01
127.95
126.91
126.87
126.82
126.74
125.96
116.39
104.14
104.12
77.33
77.07
76.82
129.62
129.28
129.03
128.80
128.01
127.95
126.91
126.87
126.82
126.74
f1 (ppm)

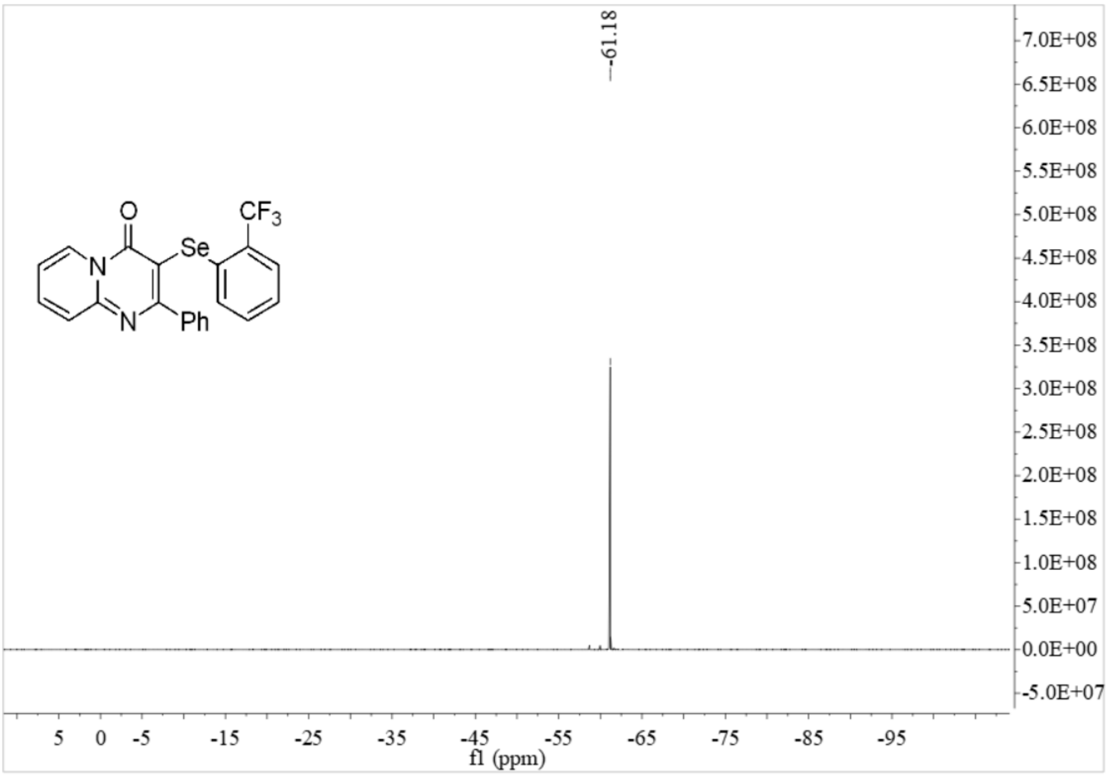

-61.18
7.0E+08
6.5E+08
6.0E+08
5.5E+08
5.0E+08
4.5E+08
4.0E+08
3.5E+08
3.0E+08
2.5E+08
2.0E+08
1.5E+08
1.0E+08
5.0E+07
0.0E+00
-5.0E+07
5 0 -5 -15 -25 -35 -45 -55 -65 -75 -85 -95
f1 (ppm)
O
CF3
N
Se
N
Ph

Compound 3m

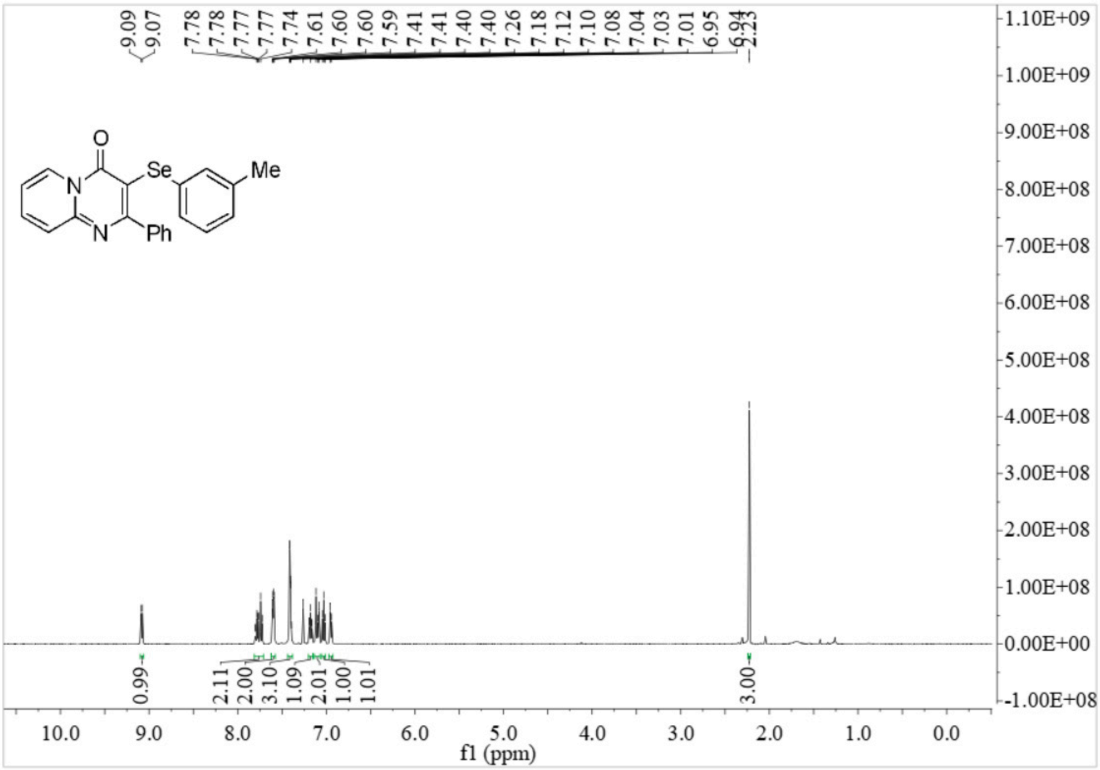

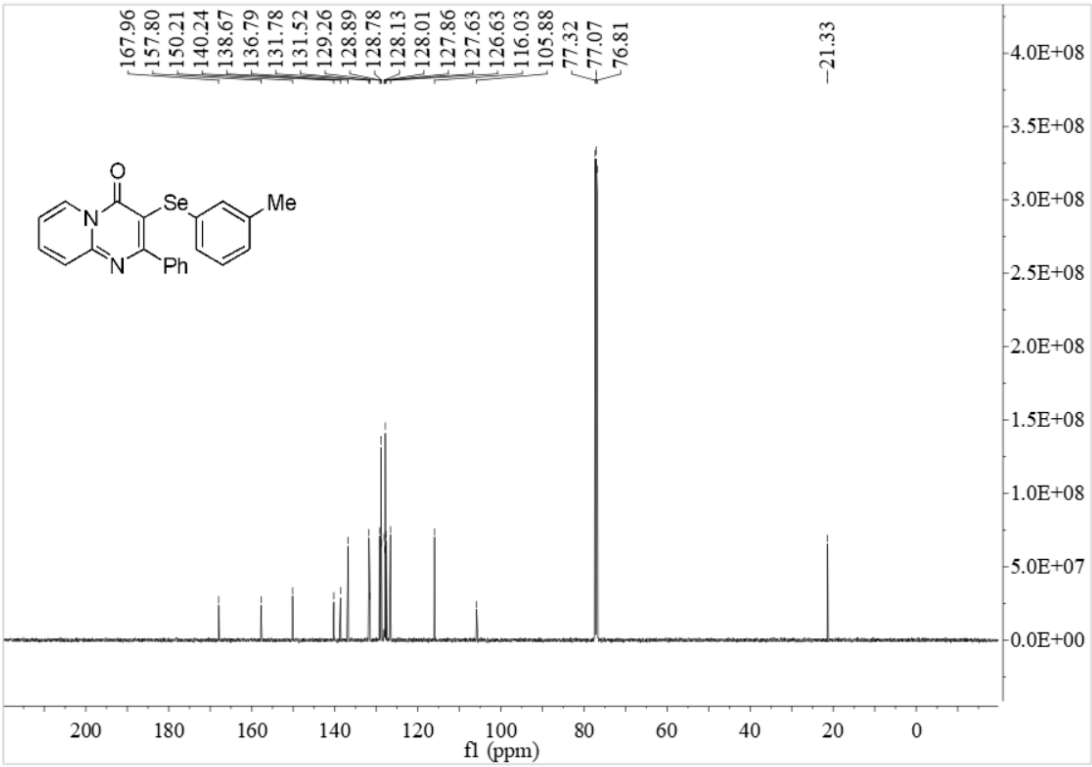

Compound 3n

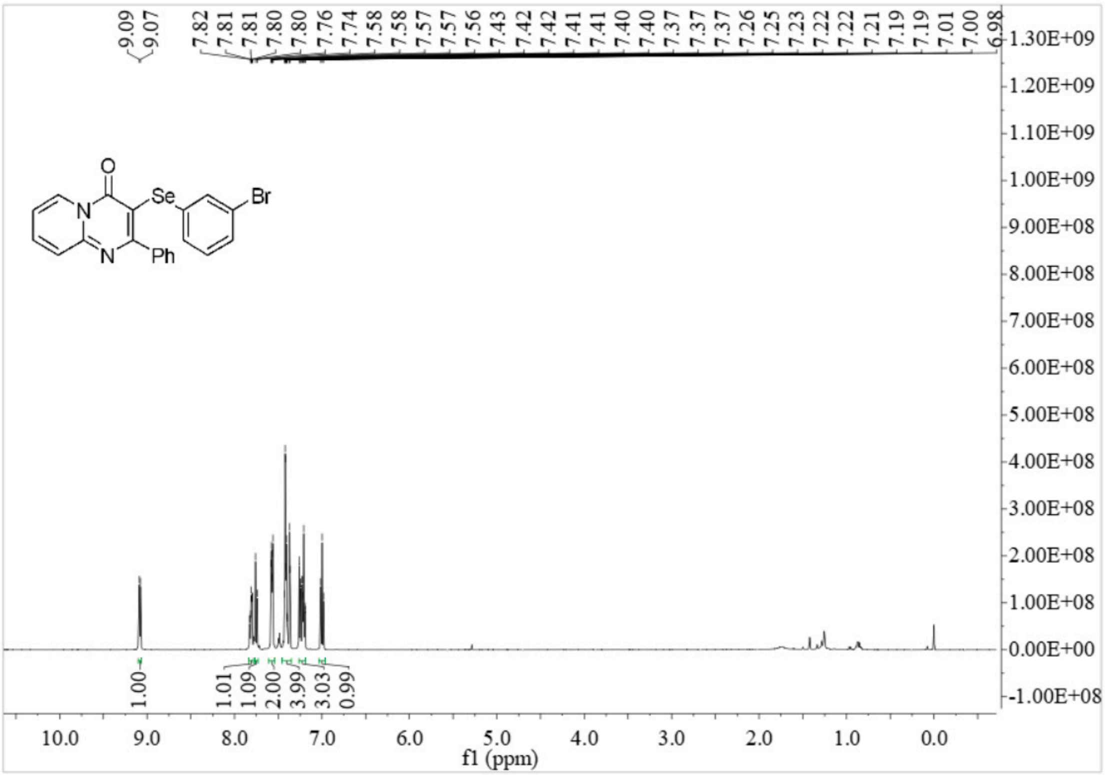
9.09
9.07
7.82
7.81
7.81
7.80
7.80
7.76
7.74
7.58
7.58
7.57
7.57
7.56
7.43
7.42
7.42
7.41
7.41
7.40
7.40
7.37
7.37
7.37
7.26
7.25
7.23
7.22
7.22
7.21
7.19
7.19
7.01
7.00
6.98
Se
Br
O
N
N
Ph
1.00
1.01
1.09
2.00
3.99
3.03
0.99
1.30E+09
1.20E+09
1.10E+09
1.00E+09
9.00E+08
8.00E+08
7.00E+08
6.00E+08
5.00E+08
4.00E+08
3.00E+08
2.00E+08
1.00E+08
0.00E+00
-1.00E+08
10.0
9.0
8.0
7.0
6.0
5.0
4.0
3.0
2.0
1.0
0.0
f1 (ppm)

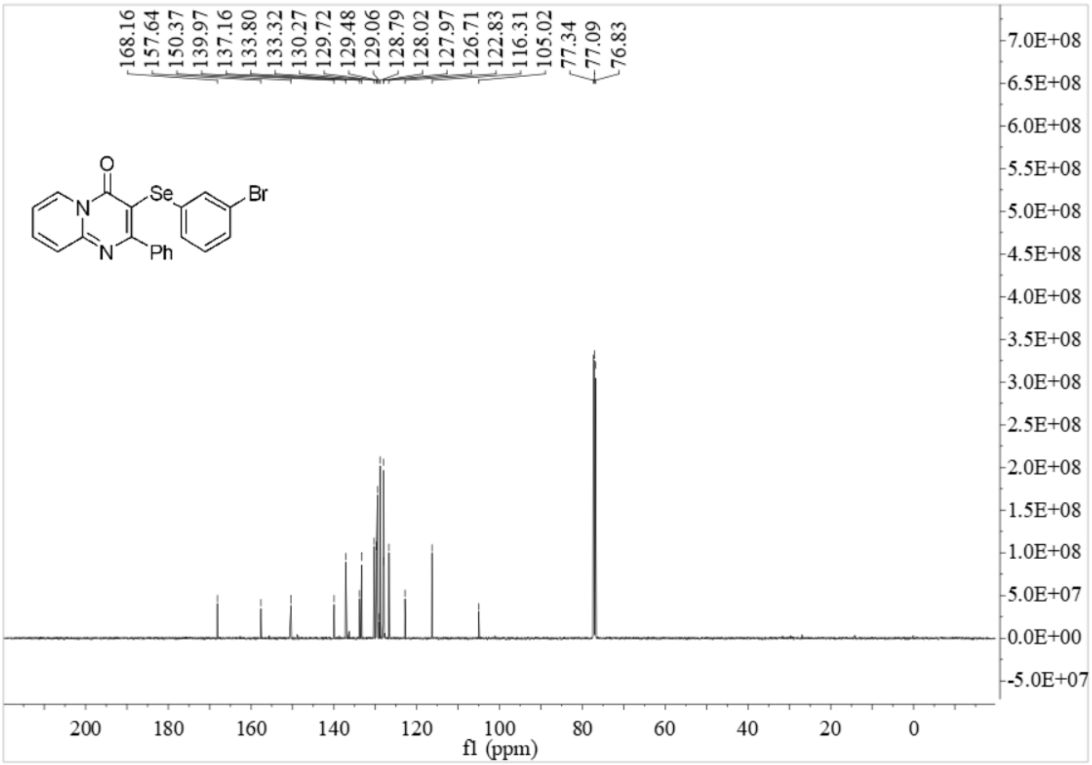
168.16
157.64
150.37
139.97
137.16
133.80
133.32
130.27
129.72
129.48
129.06
128.79
128.02
127.97
126.71
122.83
116.31
105.02
77.34
77.09
76.83
Se
Br
O
N
N
Ph
7.0E+08
6.5E+08
6.0E+08
5.5E+08
5.0E+08
4.5E+08
4.0E+08
3.5E+08
3.0E+08
2.5E+08
2.0E+08
1.5E+08
1.0E+08
5.0E+07
0.0E+00
-5.0E+07
200
180
160
140
120
100
80
60
40
20
0
f1 (ppm)

Compound 3o

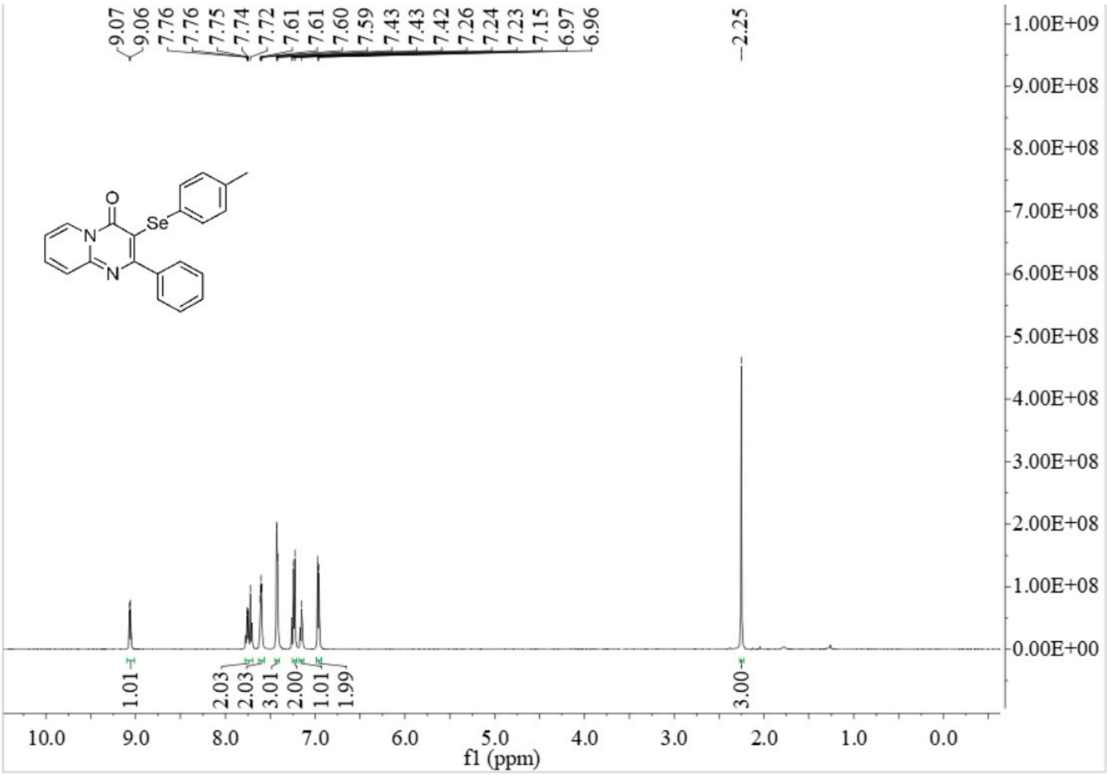
9.07
9.06
7.76
7.76
7.75
7.74
7.72
7.61
7.61
7.60
7.59
7.43
7.43
7.42
7.26
7.24
7.23
7.15
6.97
6.96
2.25
Se
O
N
1.01
2.03
2.03
3.01
2.00
1.01
1.99
3.00
10.0
9.0
8.0
7.0
6.0
5.0
4.0
3.0
2.0
1.0
0.0
f1 (ppm)

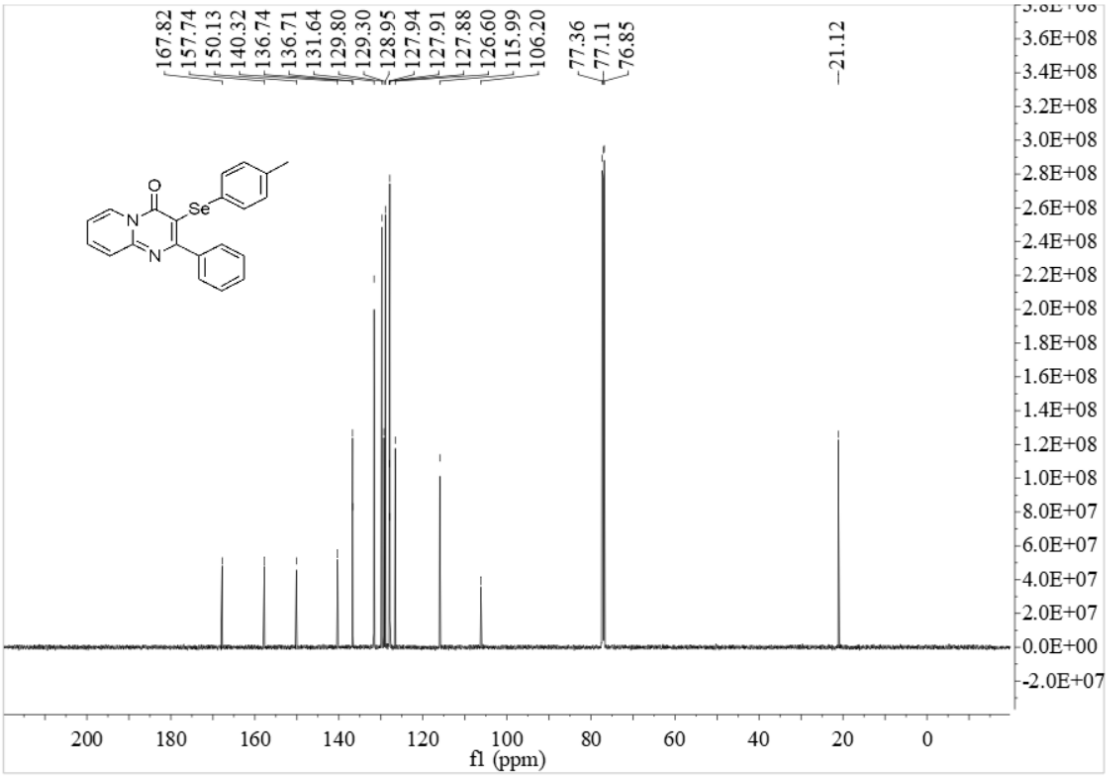
167.82
157.74
150.13
140.32
136.74
136.71
131.64
129.80
129.30
128.95
127.94
127.91
127.88
126.60
115.99
106.20
77.36
77.11
76.85
21.12
Se
O
N
200
180
160
140
120
100
80
60
40
20
0
f1 (ppm)

Compound 3p

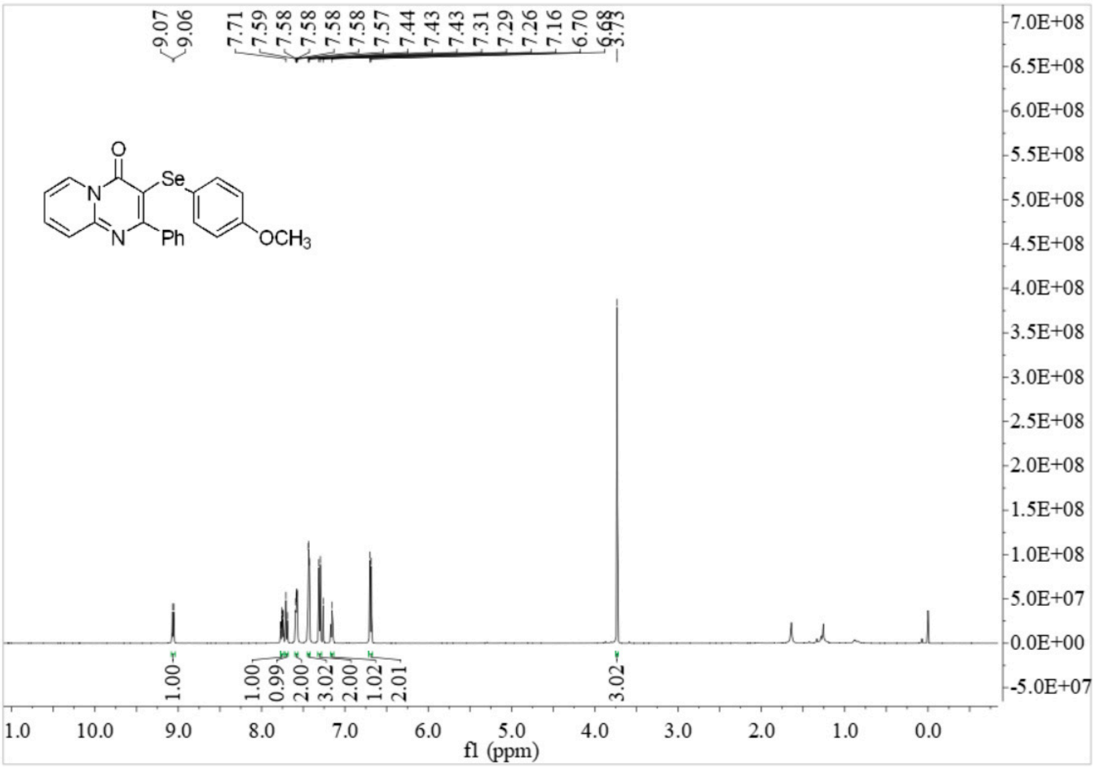
9.07
9.06
7.71
7.59
7.58
7.58
7.58
7.58
7.57
7.44
7.43
7.43
7.31
7.29
7.26
7.16
6.70
6.68
3.73
Se
N
O
N
Ph
OCH3
1.00
1.00
0.99
2.00
3.02
2.00
1.02
2.01
3.02
7.0E+08
6.5E+08
6.0E+08
5.5E+08
5.0E+08
4.5E+08
4.0E+08
3.5E+08
3.0E+08
2.5E+08
2.0E+08
1.5E+08
1.0E+08
5.0E+07
0.0E+00
-5.0E+07
1.0
10.0
9.0
8.0
7.0
6.0
5.0
4.0
3.0
2.0
1.0
0.0
f1 (ppm)

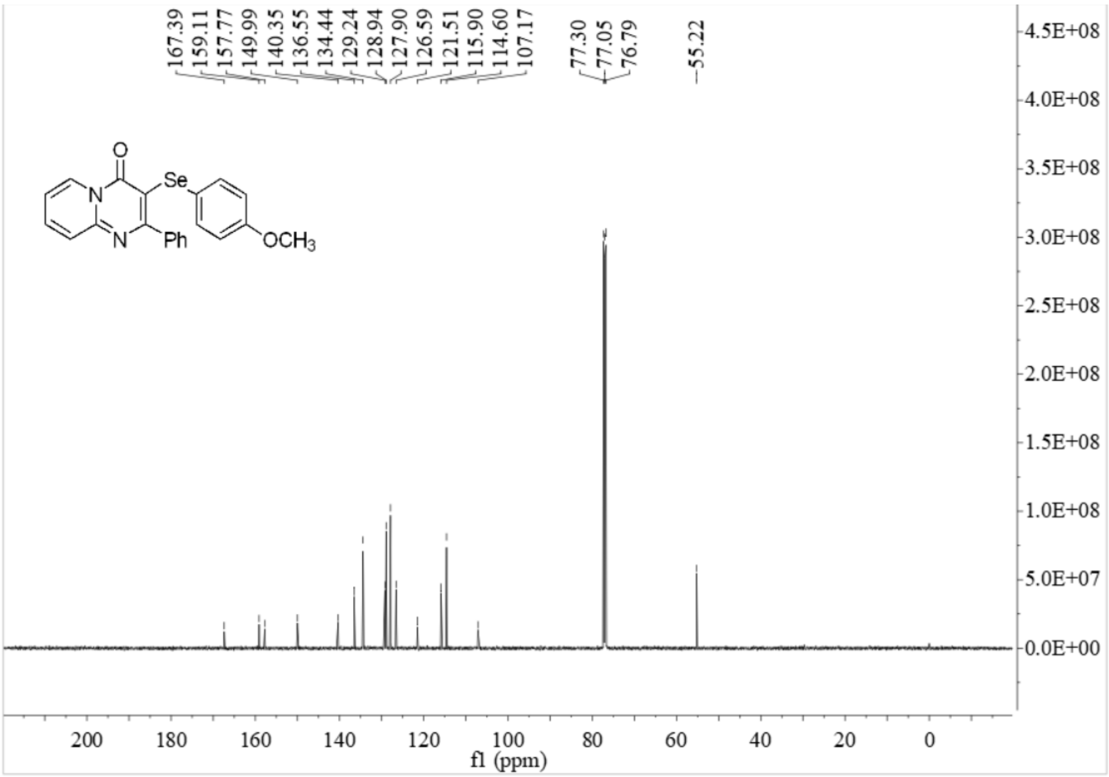
167.39
159.11
157.77
149.99
140.35
136.55
134.44
129.24
128.94
127.90
126.59
121.51
115.90
114.60
107.17
77.30
77.05
76.79
55.22
Se
N
O
N
Ph
OCH3
4.5E+08
4.0E+08
3.5E+08
3.0E+08
2.5E+08
2.0E+08
1.5E+08
1.0E+08
5.0E+07
0.0E+00
200
180
160
140
120
100
80
60
40
20
0
f1 (ppm)

Compound 3q

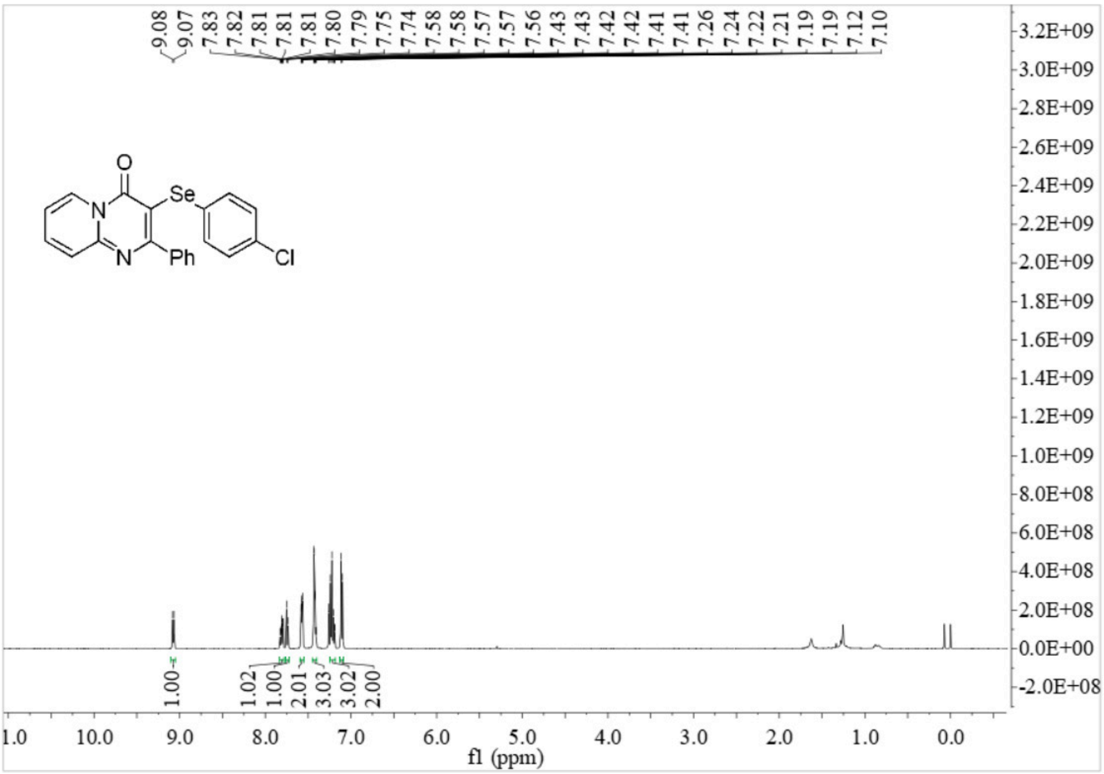

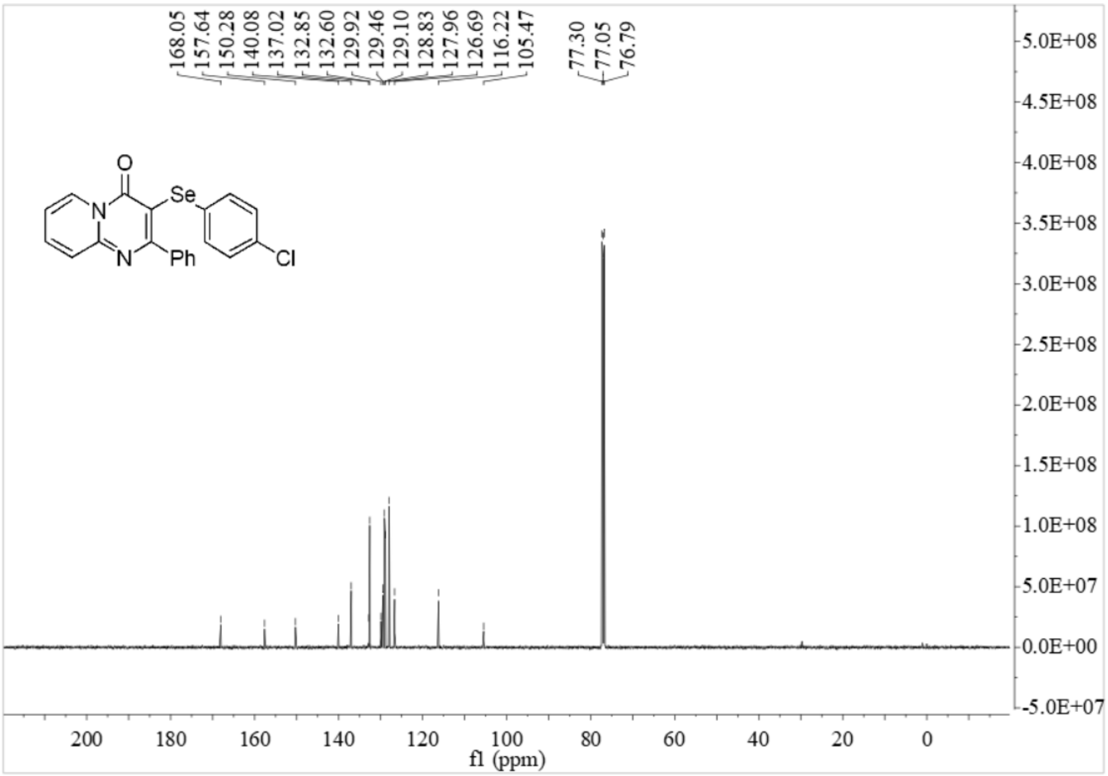

Compound 3r

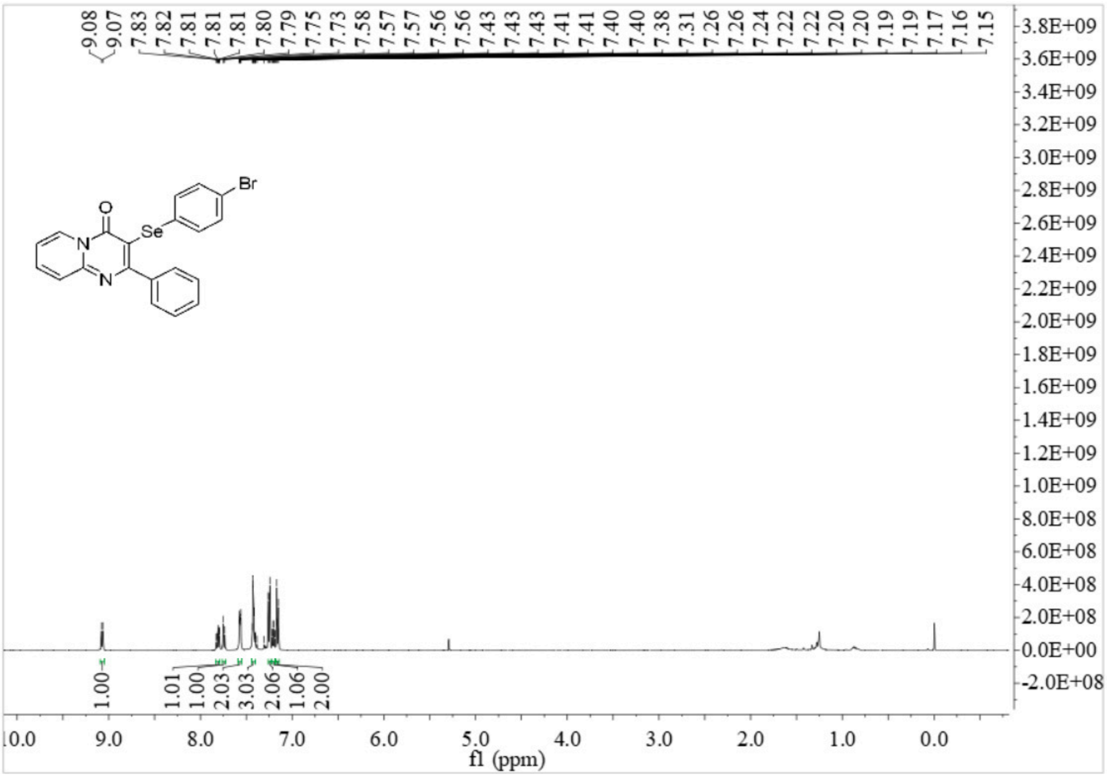

9.08
9.07
7.83
7.82
7.81
7.81
7.81
7.80
7.79
7.75
7.73
7.58
7.57
7.57
7.56
7.56
7.43
7.43
7.43
7.41
7.41
7.40
7.40
7.38
7.31
7.26
7.26
7.24
7.22
7.22
7.20
7.20
7.19
7.19
7.17
7.16
7.15
Br
O
Se
N
N
1.00
1.01
1.00
2.03
3.03
2.06
1.06
2.00
f1 (ppm)

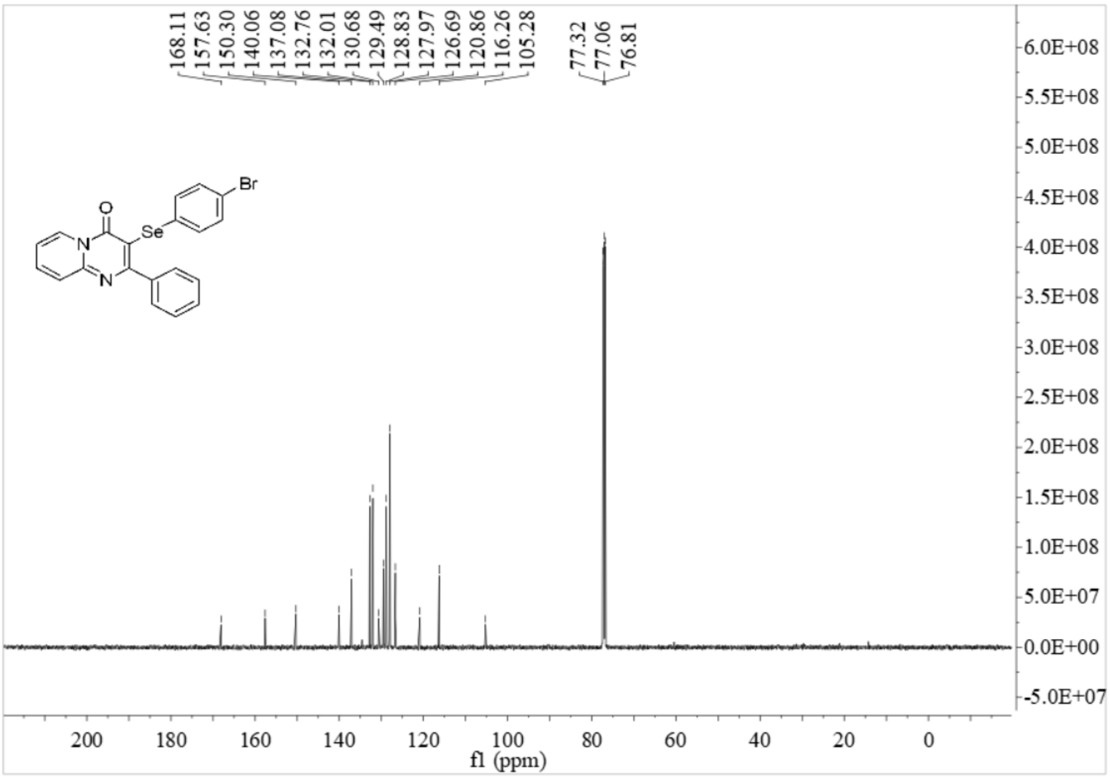

168.11
157.63
150.30
140.06
137.08
132.76
132.01
130.68
129.49
128.83
127.97
126.69
120.86
116.26
105.28
77.32
77.06
76.81
Br
O
Se
N
N
f1 (ppm)

Compound 3s

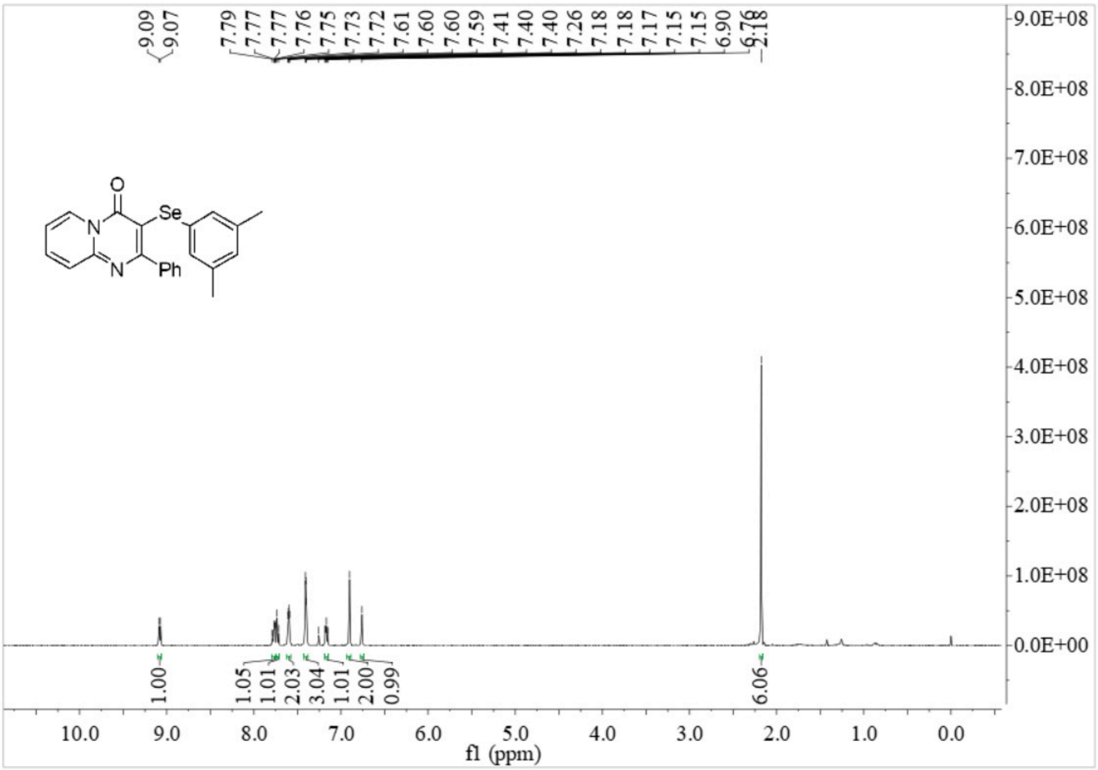

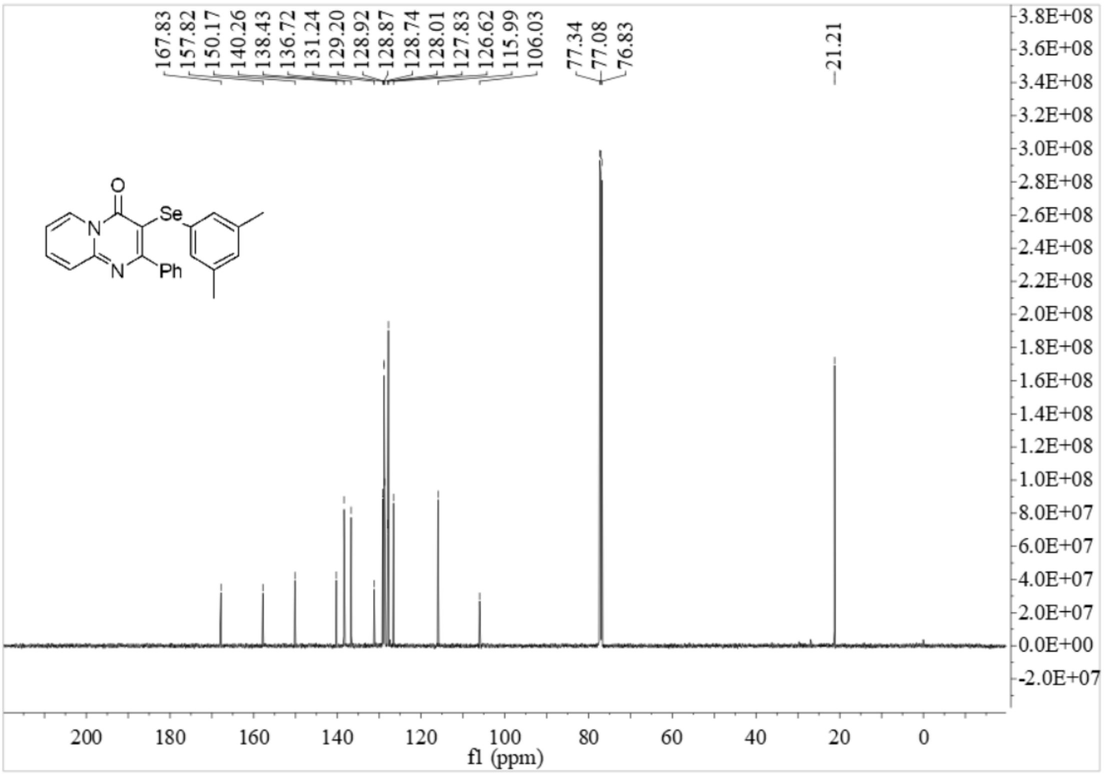

Compound 3t

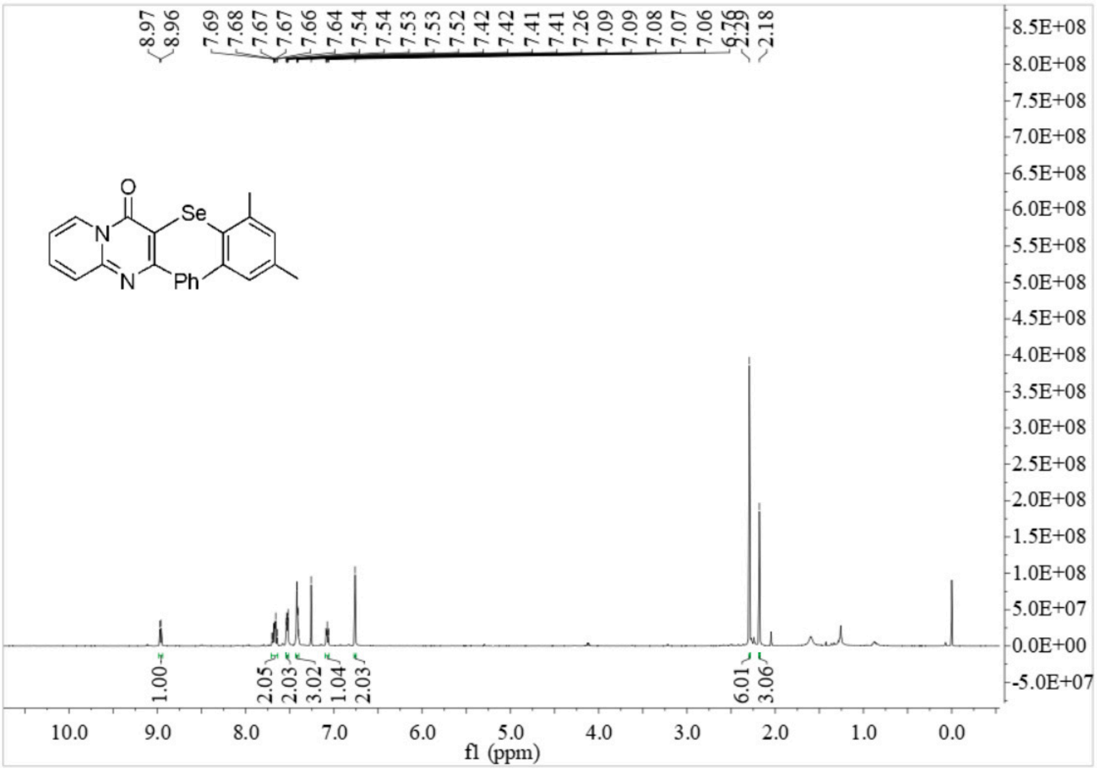

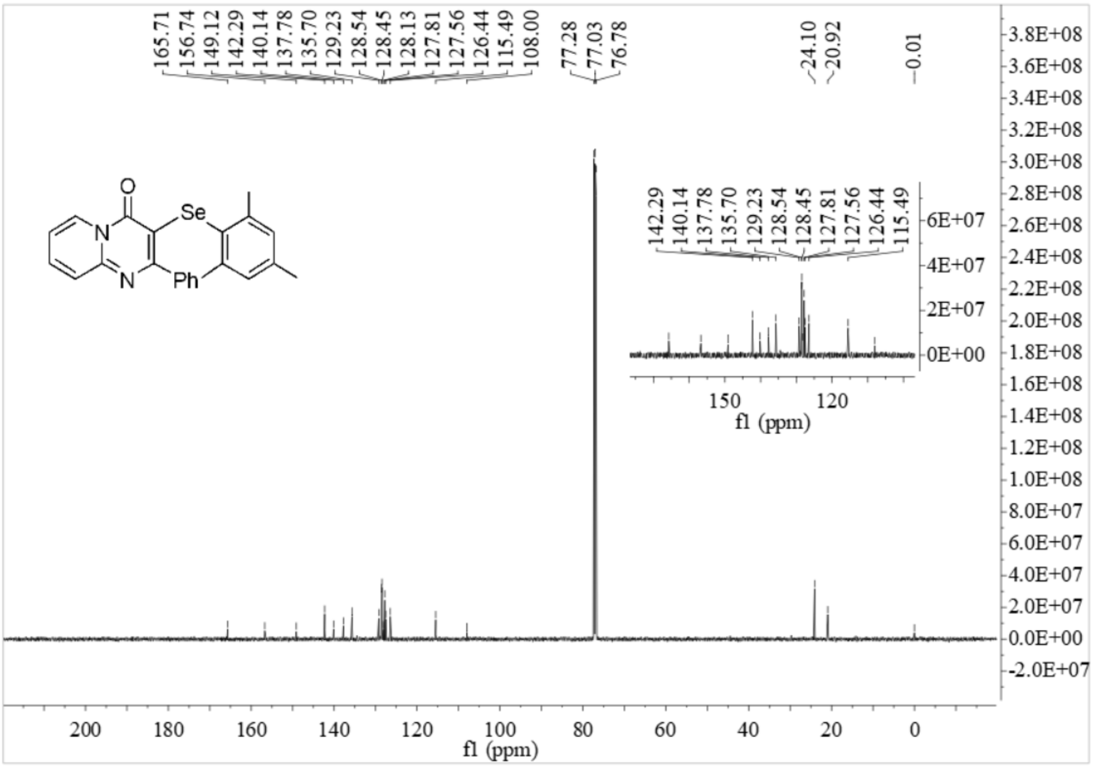

Compound 3u

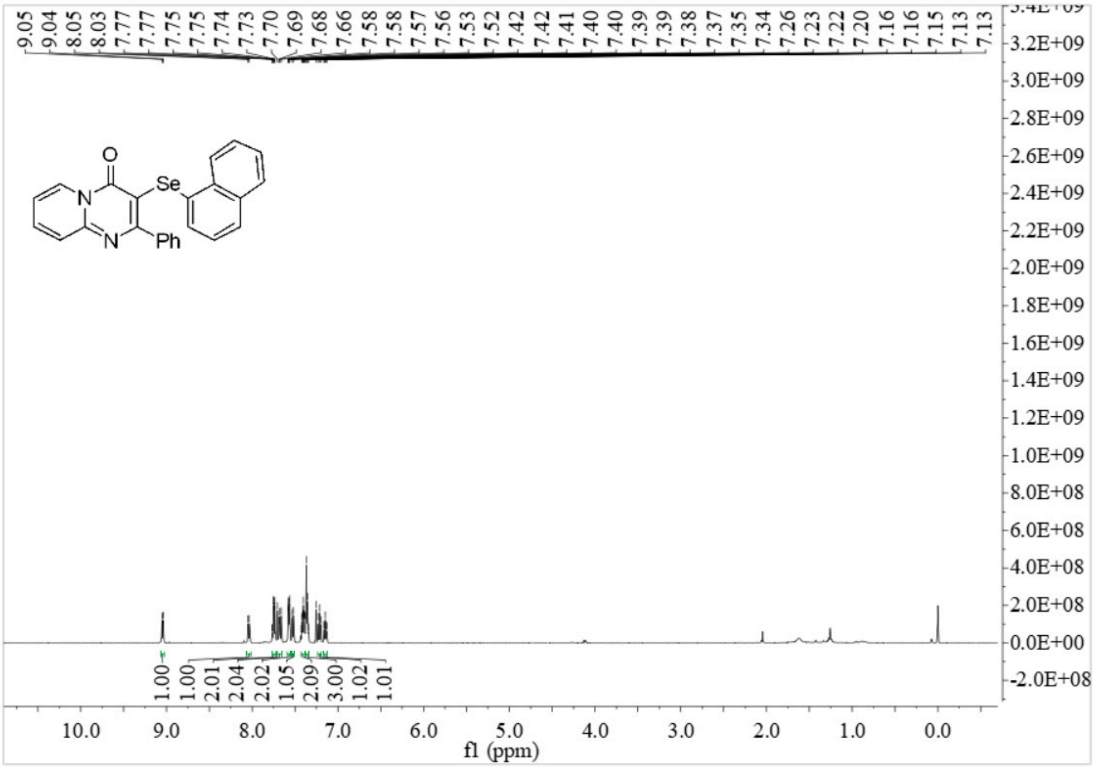

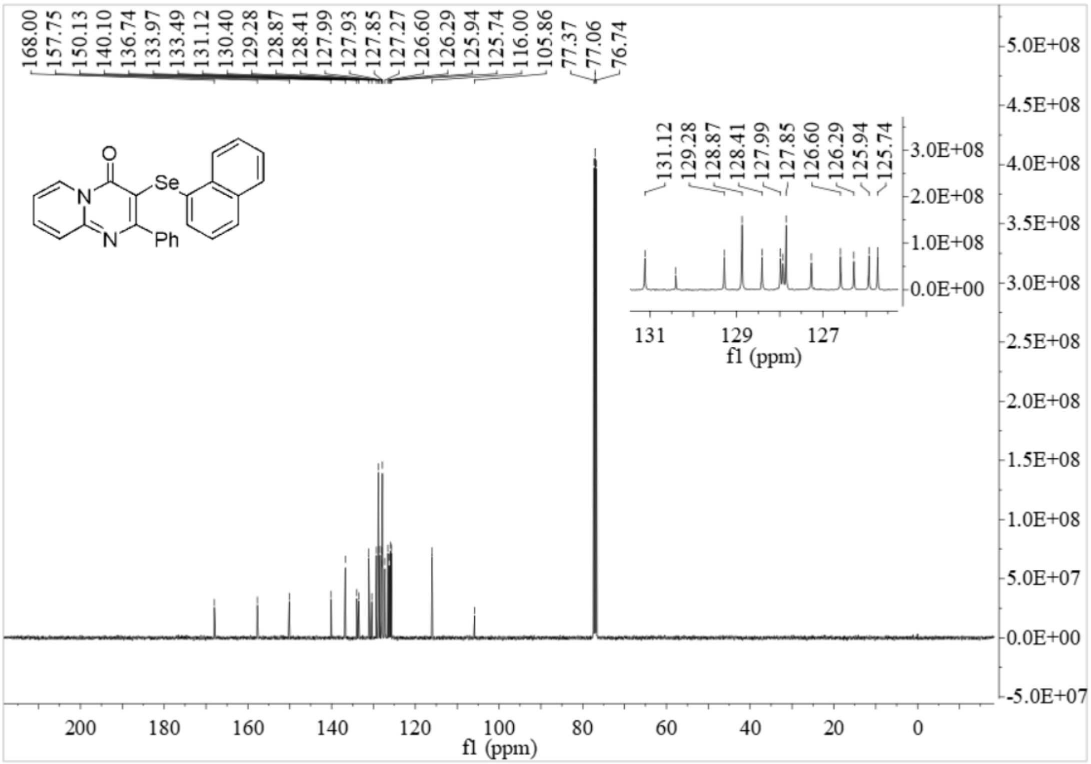

Compound 3v

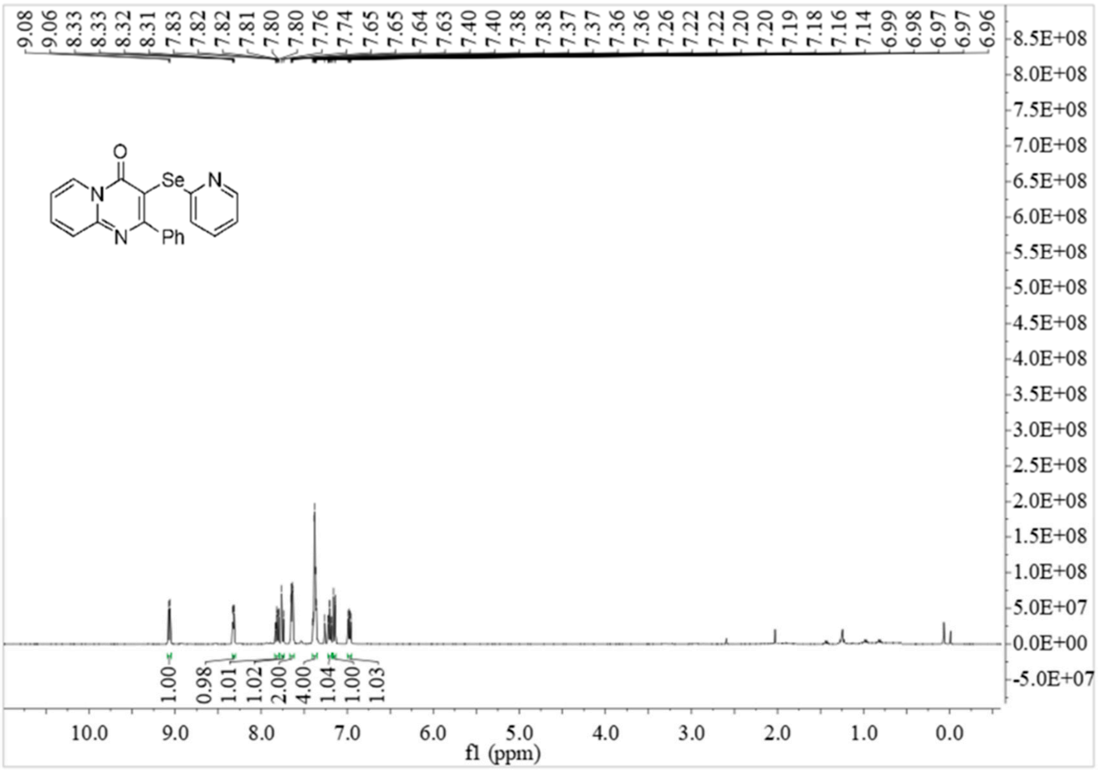
9.08
9.06
8.33
8.33
8.32
8.31
7.83
7.82
7.82
7.81
7.80
7.80
7.76
7.74
7.65
7.65
7.64
7.63
7.40
7.40
7.38
7.38
7.37
7.37
7.36
7.36
7.26
7.22
7.22
7.20
7.20
7.19
7.18
7.16
7.14
6.99
6.98
6.97
6.97
6.96
O
Se
N
N
N
Ph
1.00
0.98
1.01
1.02
2.00
4.00
1.04
1.00
1.03
fl (ppm)

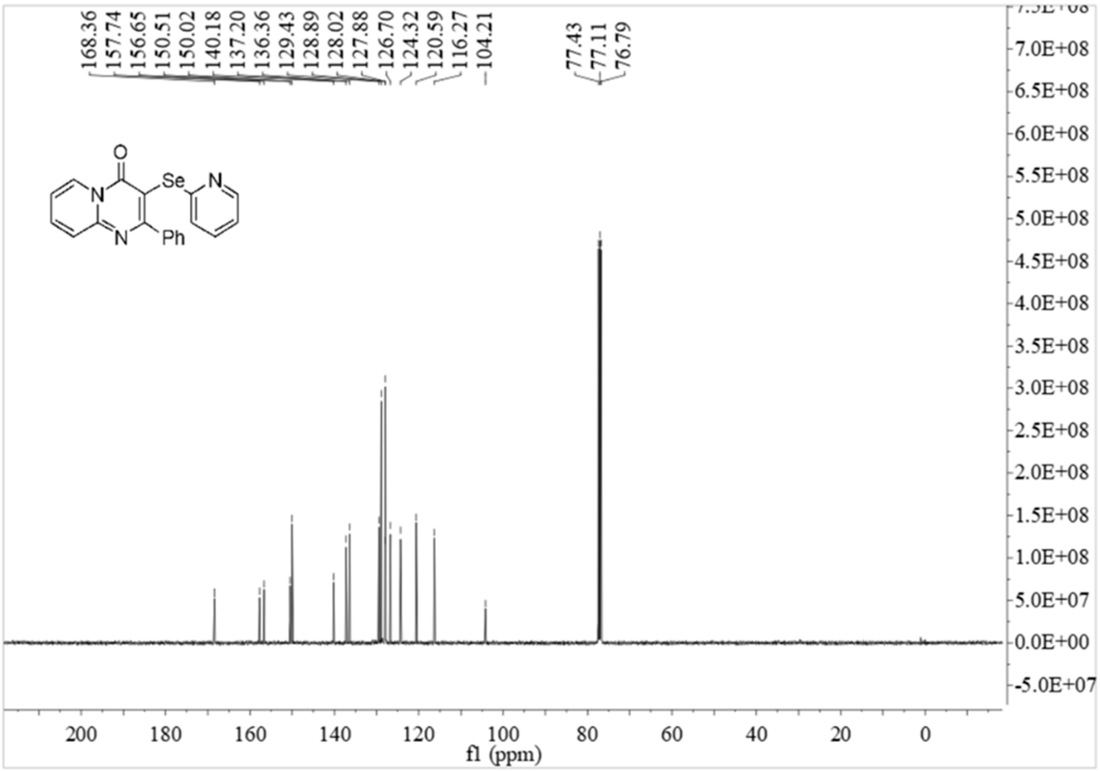
168.36
157.74
156.65
150.51
150.02
140.18
137.20
136.36
129.43
128.89
128.02
127.88
126.70
124.32
120.59
116.27
104.21
77.43
77.11
76.79
O
Se
N
N
N
Ph
fl (ppm)

Compound 3w

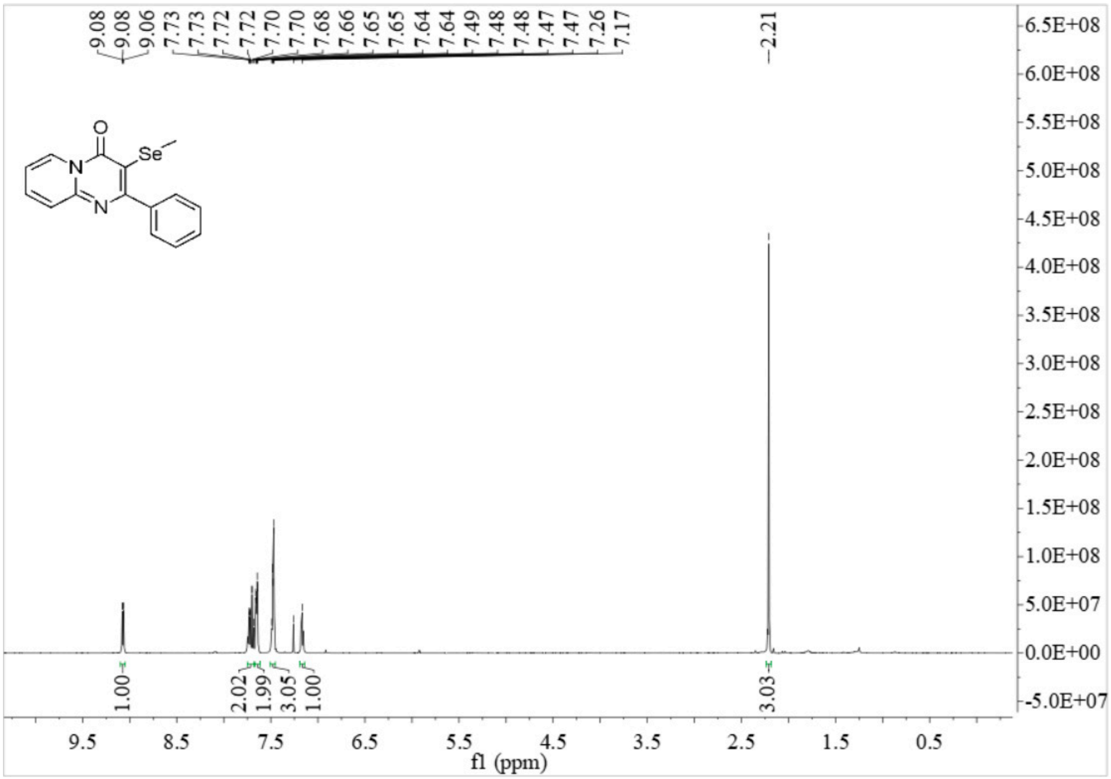
9.08
9.08
9.06
7.73
7.73
7.72
7.72
7.70
7.70
7.68
7.66
7.65
7.65
7.64
7.64
7.49
7.48
7.48
7.47
7.47
7.26
7.17
2.21
O
Se
N
N
1.00
2.02
1.99
3.05
1.00
3.03
6.5E+08
6.0E+08
5.5E+08
5.0E+08
4.5E+08
4.0E+08
3.5E+08
3.0E+08
2.5E+08
2.0E+08
1.5E+08
1.0E+08
5.0E+07
0.0E+00
-5.0E+07
9.5
8.5
7.5
6.5
5.5
4.5
3.5
2.5
1.5
0.5
f1 (ppm)

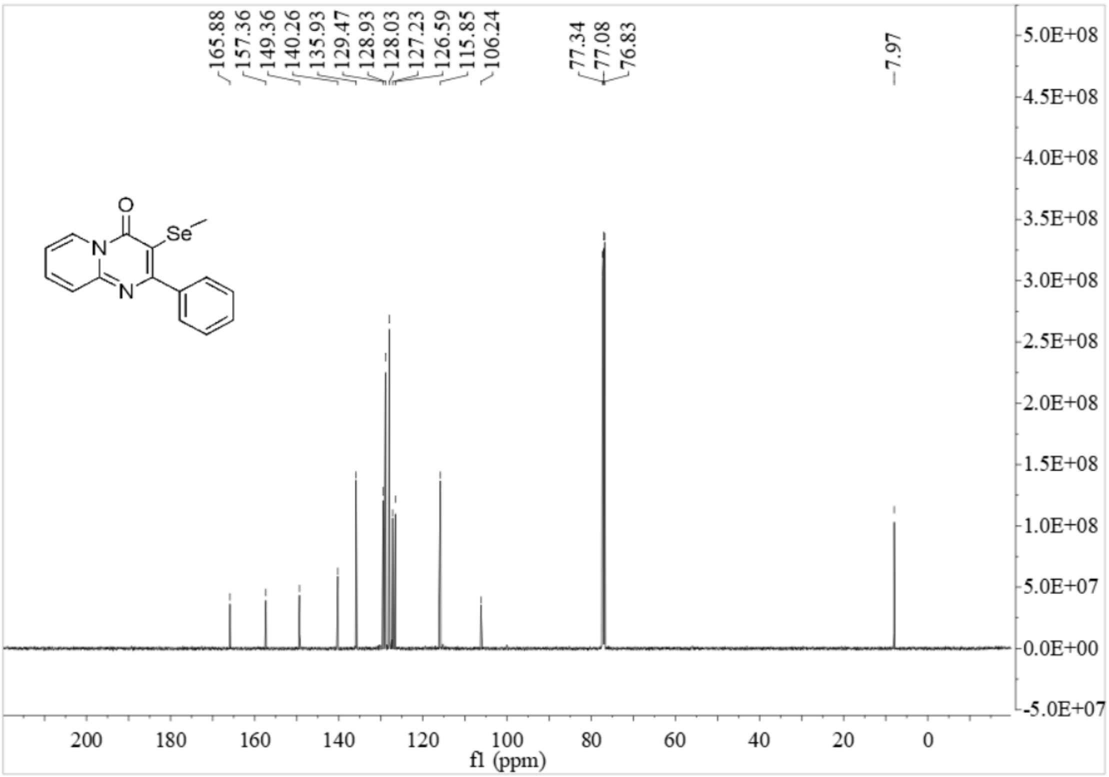
165.88
157.36
149.36
140.26
135.93
129.47
128.93
128.03
127.23
126.59
115.85
106.24
77.34
77.08
76.83
7.97
O
Se
N
N
5.0E+08
4.5E+08
4.0E+08
3.5E+08
3.0E+08
2.5E+08
2.0E+08
1.5E+08
1.0E+08
5.0E+07
0.0E+00
-5.0E+07
200
180
160
140
120
100
80
60
40
20
0
f1 (ppm)

Compound 3x

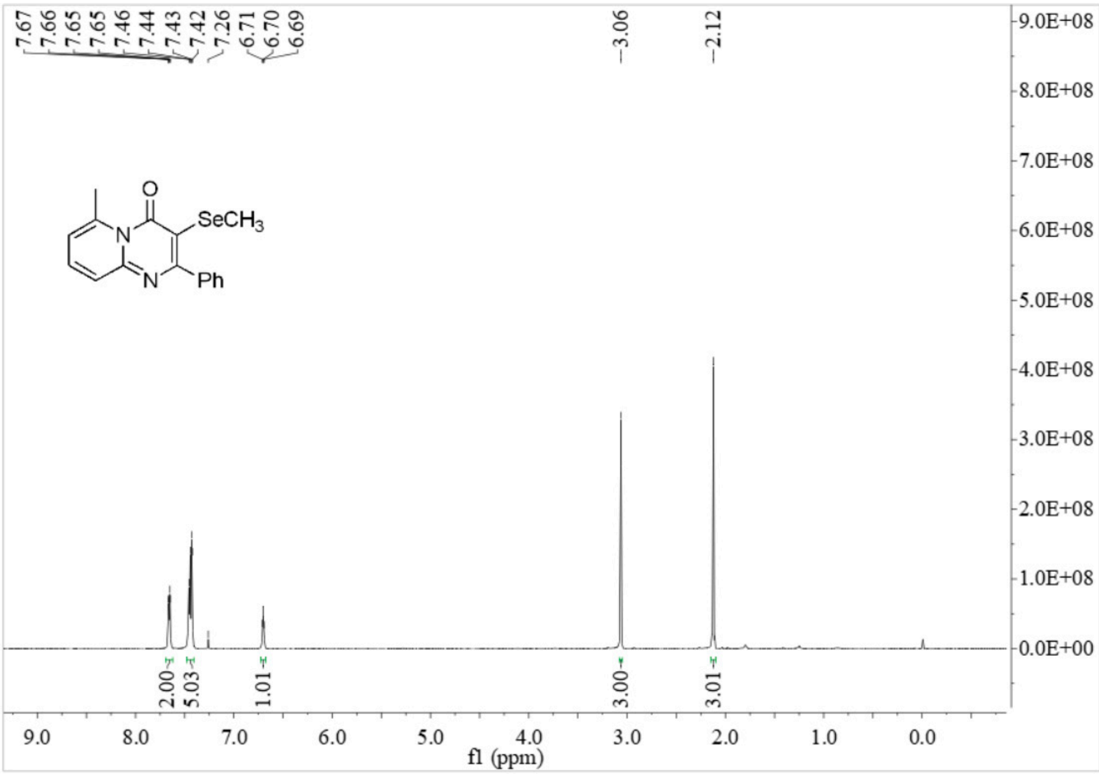

7.67
7.66
7.65
7.65
7.46
7.44
7.43
7.42
7.26
6.71
6.70
6.69
3.06
2.12
SeCH3
N
O
N
Ph
2.00
5.03
1.01
3.00
3.01
9.0E+08
8.0E+08
7.0E+08
6.0E+08
5.0E+08
4.0E+08
3.0E+08
2.0E+08
1.0E+08
0.0E+00
9.0 8.0 7.0 6.0 5.0 4.0 3.0 2.0 1.0 0.0
f1 (ppm)

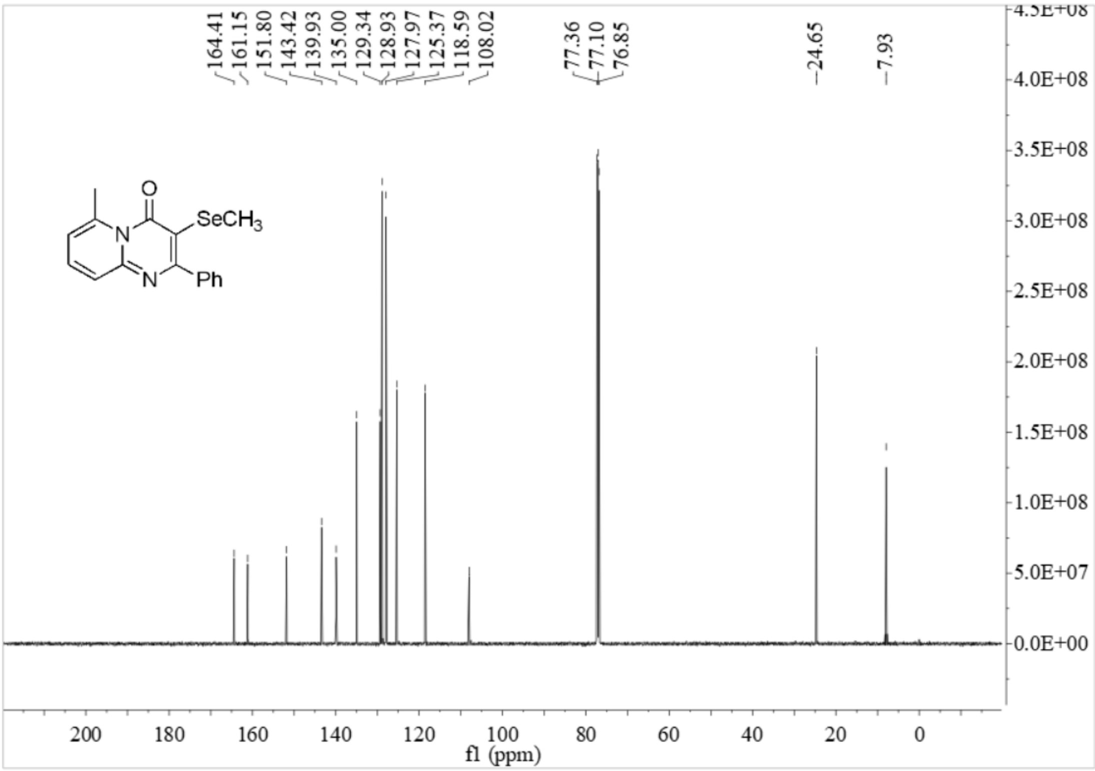

164.41
161.15
151.80
143.42
139.93
135.00
129.34
128.93
127.97
125.37
118.59
108.02
77.36
77.10
76.85
24.65
7.93
SeCH3
N
O
N
Ph
4.0E+08
3.5E+08
3.0E+08
2.5E+08
2.0E+08
1.5E+08
1.0E+08
5.0E+07
0.0E+00
200 180 160 140 120 100 80 60 40 20 0
f1 (ppm)

Compound 3y

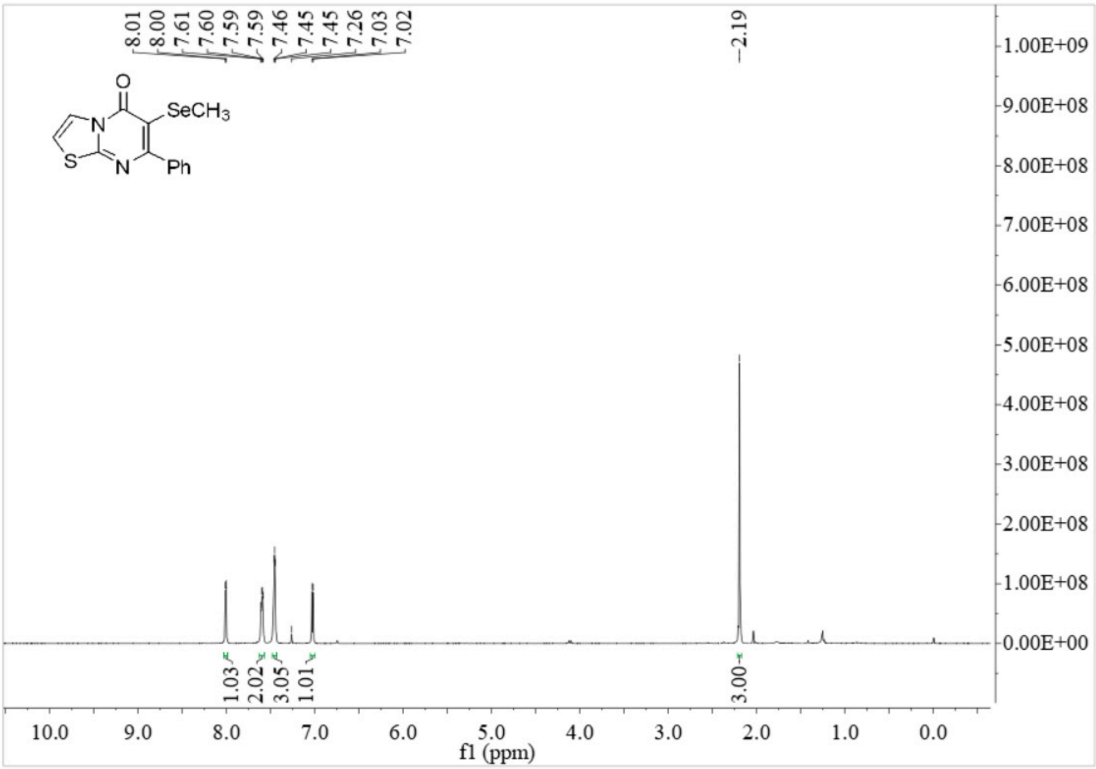

8.01
8.00
7.61
7.60
7.59
7.59
7.46
7.45
7.45
7.26
7.03
7.02
2.19
SeCH3
Ph
1.03
2.02
3.05
1.01
3.00
f1 (ppm)

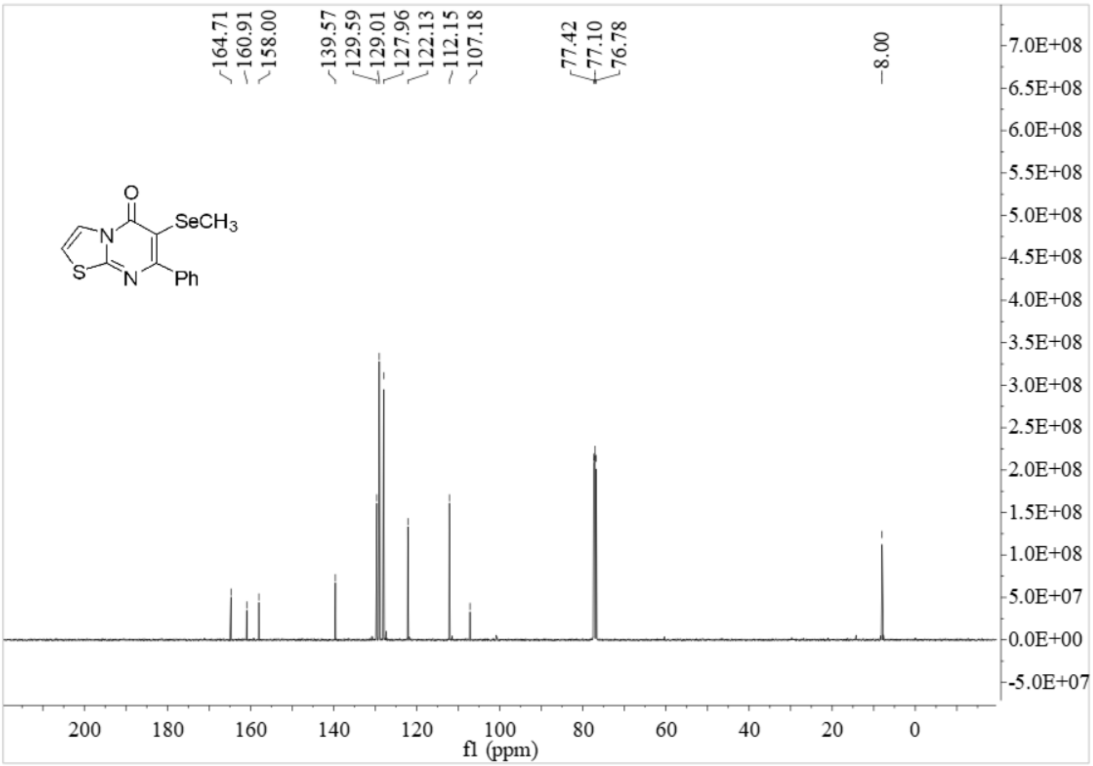

164.71
160.91
158.00
139.57
129.59
129.01
127.96
122.13
112.15
107.18
77.42
77.10
76.78
8.00
SeCH3
Ph
f1 (ppm)

Compound 3z

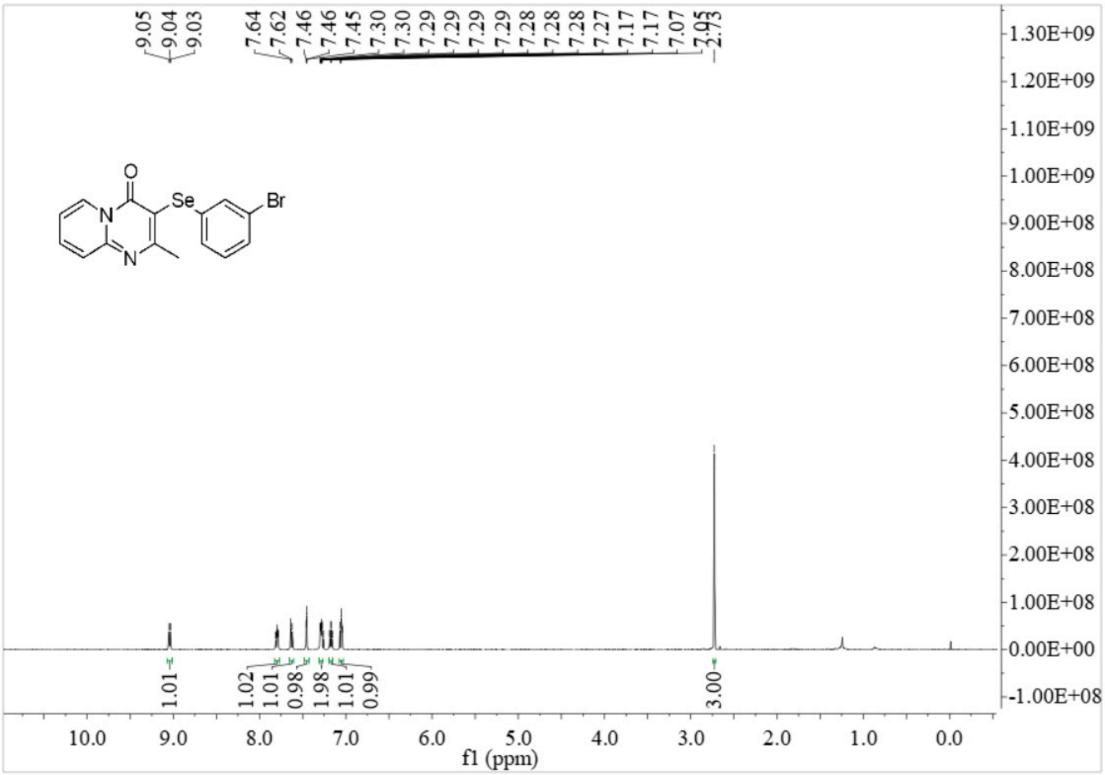

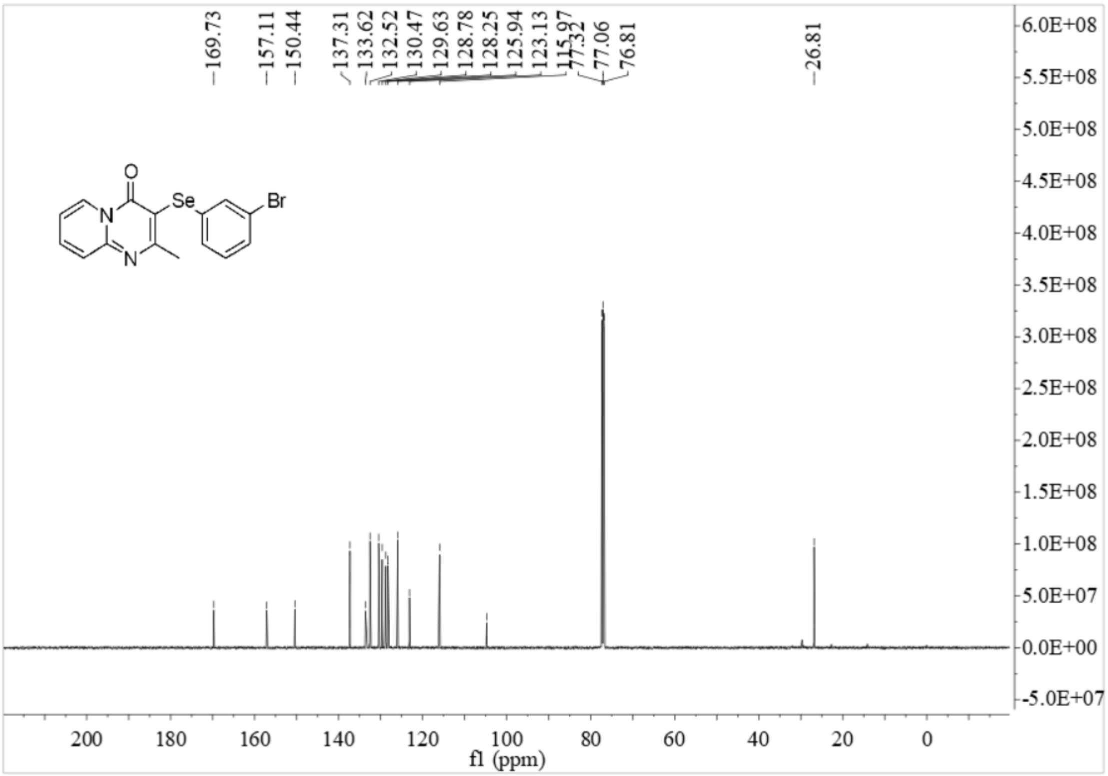

Compound 3aa

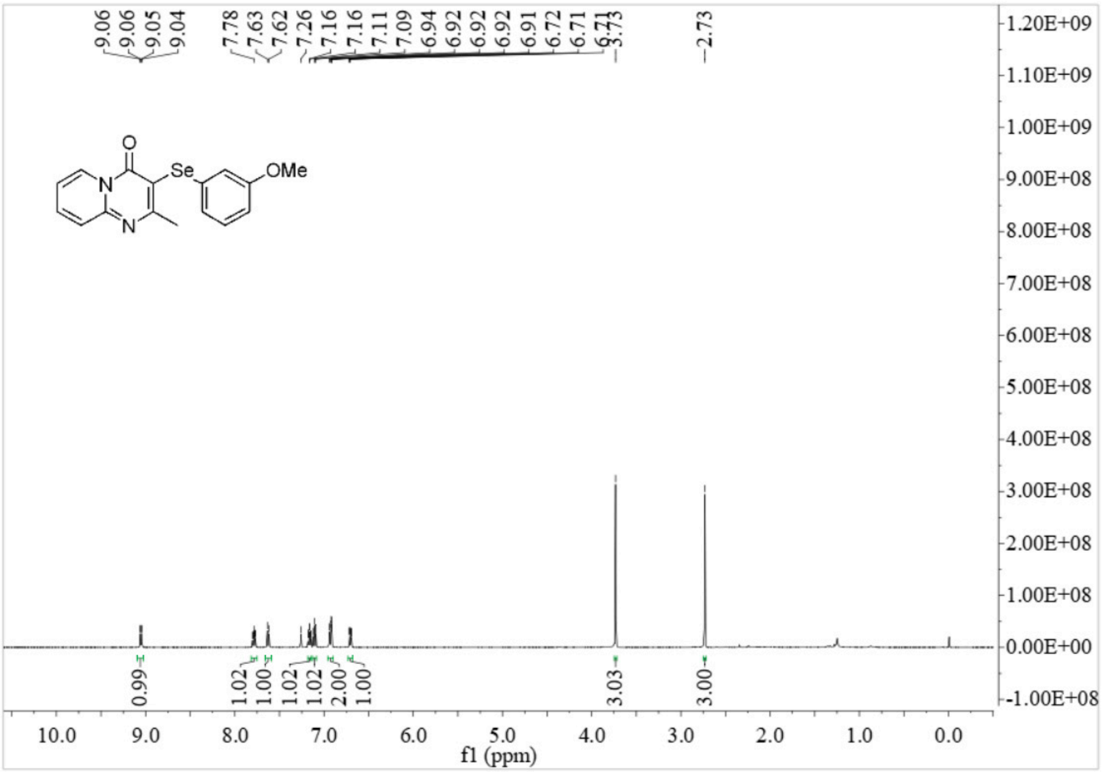

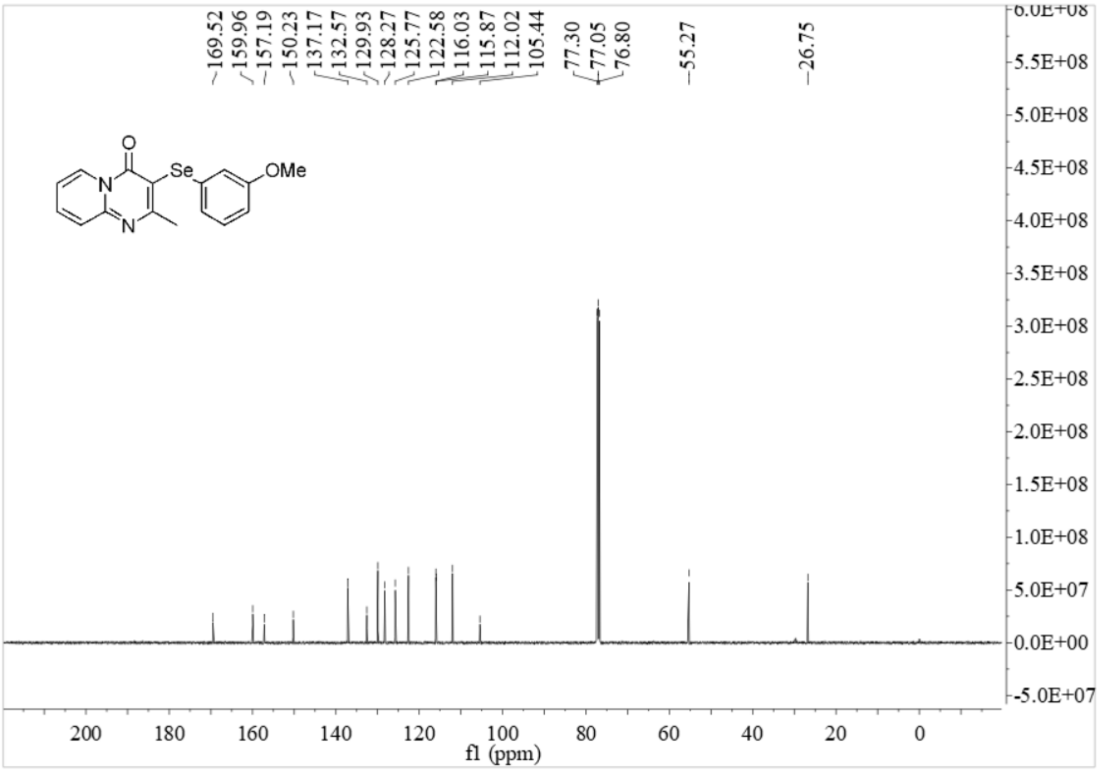

Compound 3ab

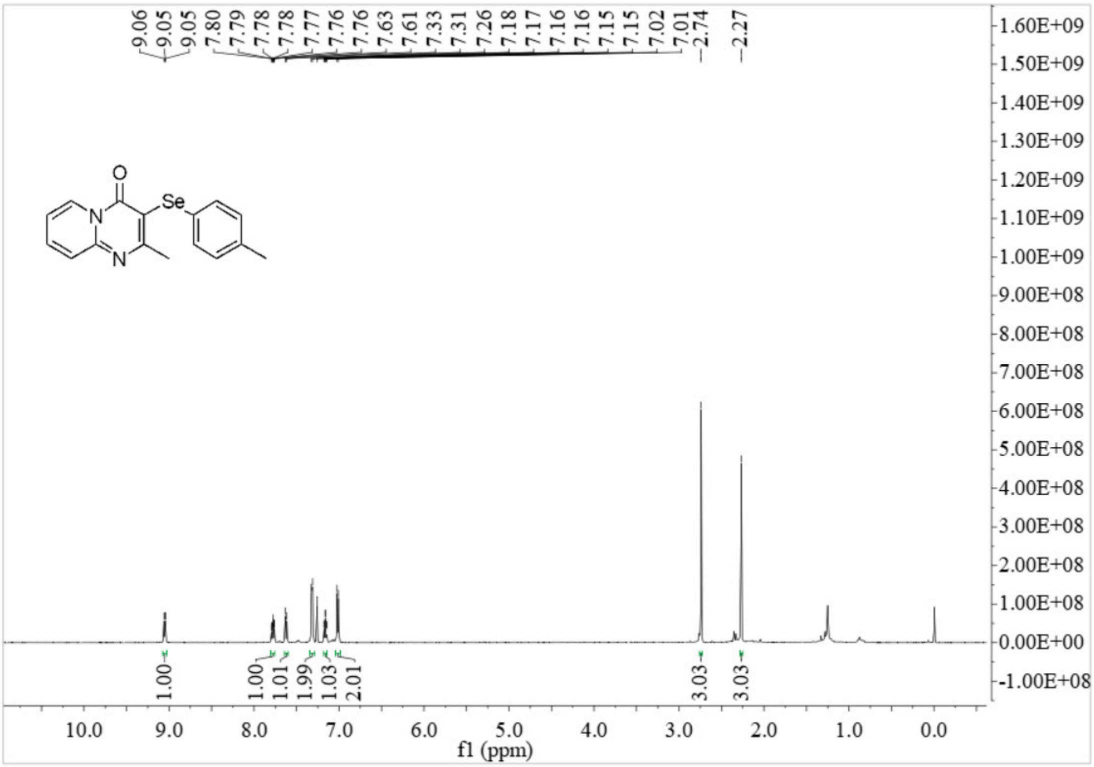

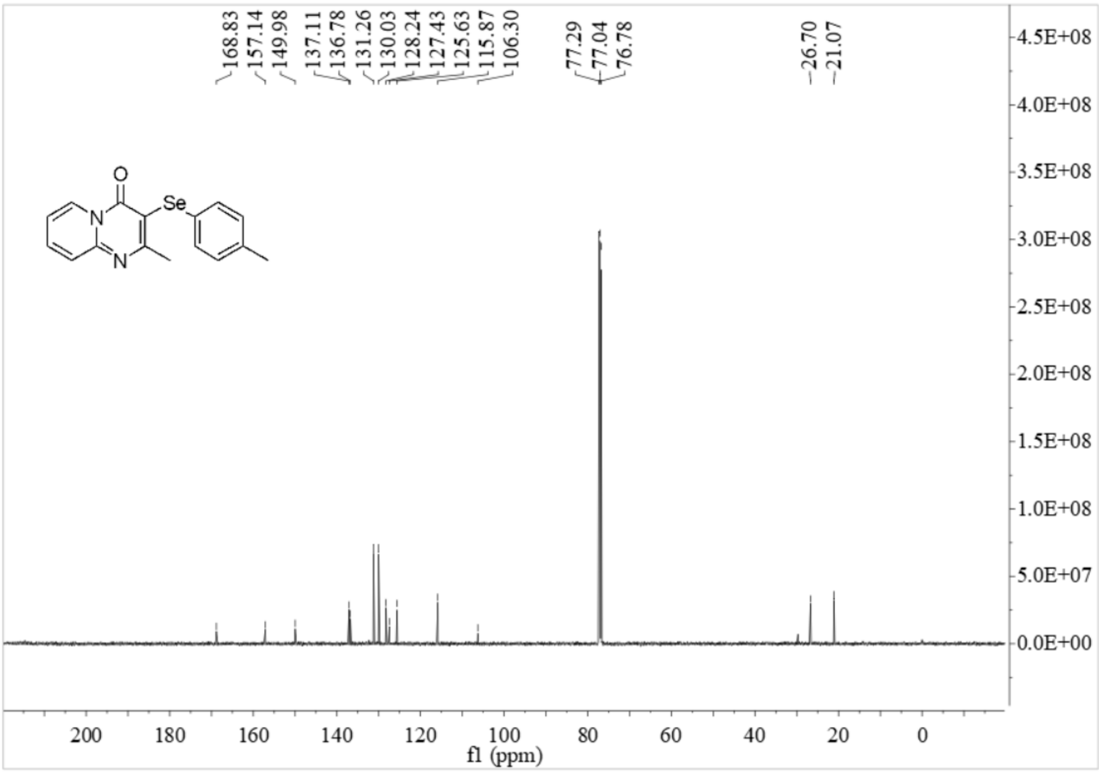

Compound 3ac

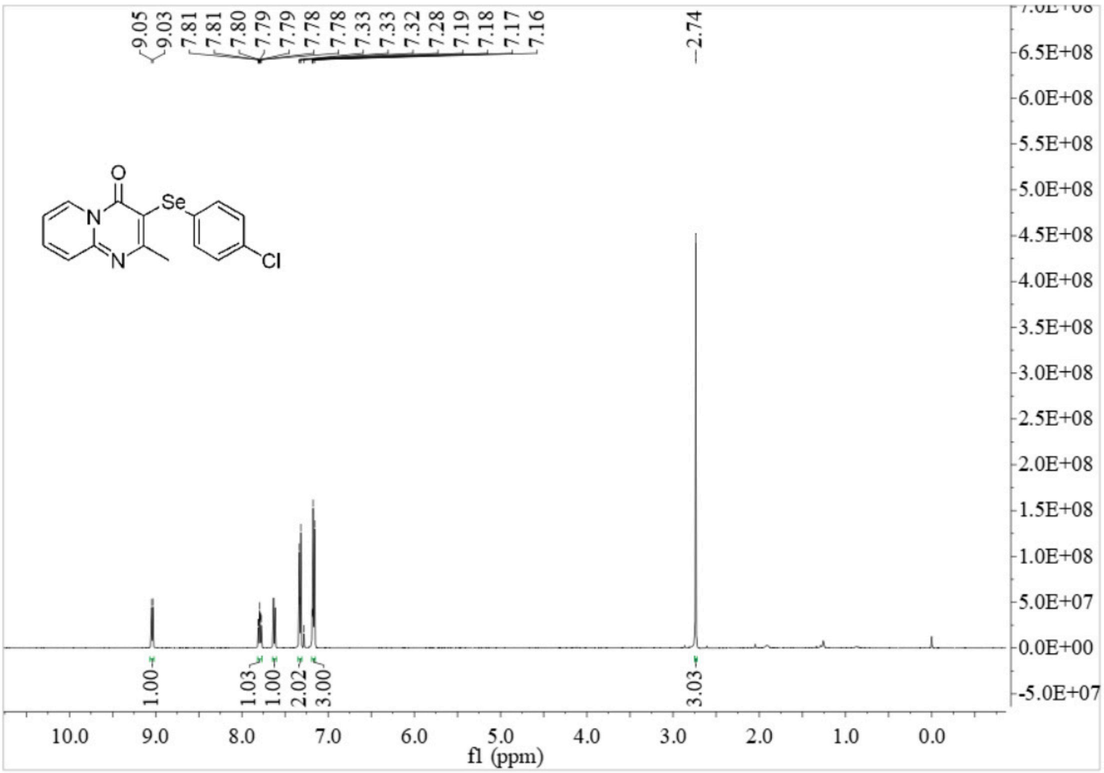

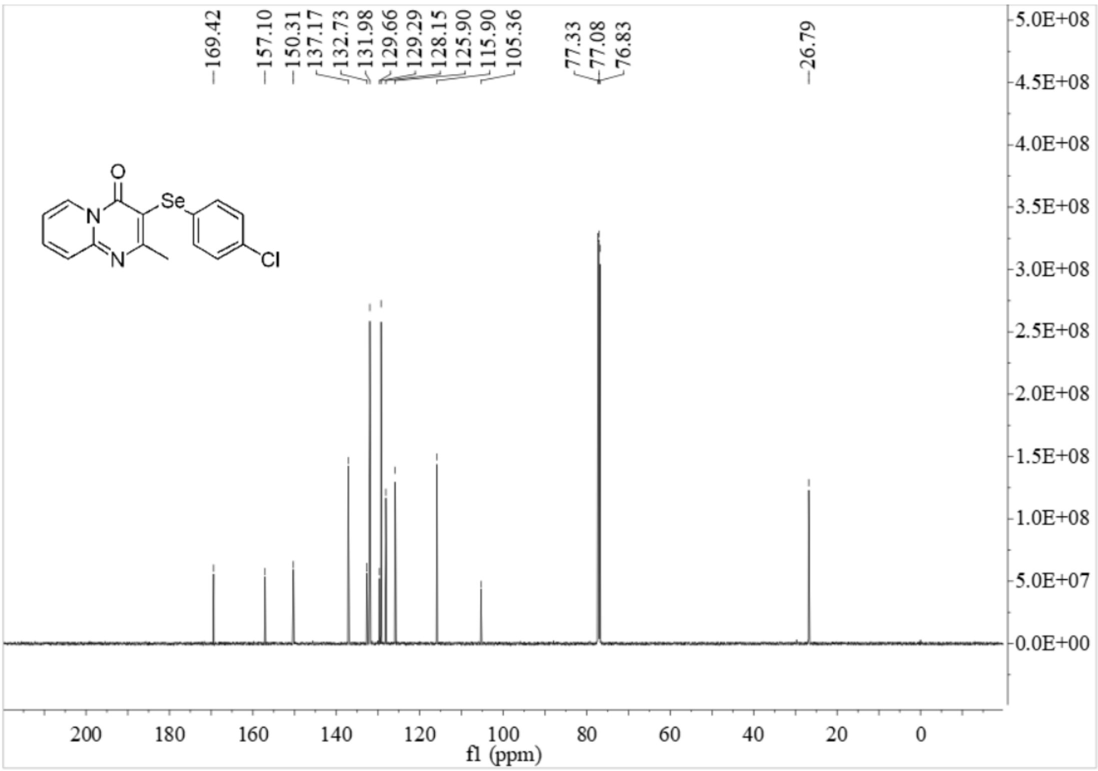

Compound 3ad

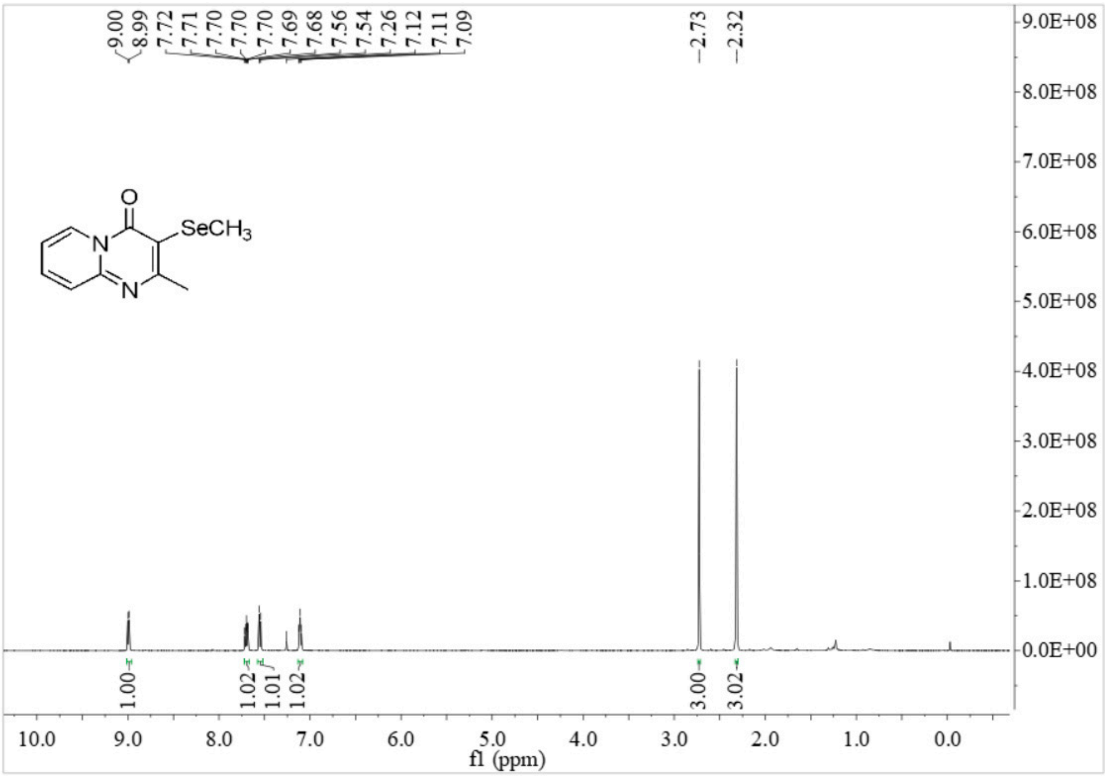

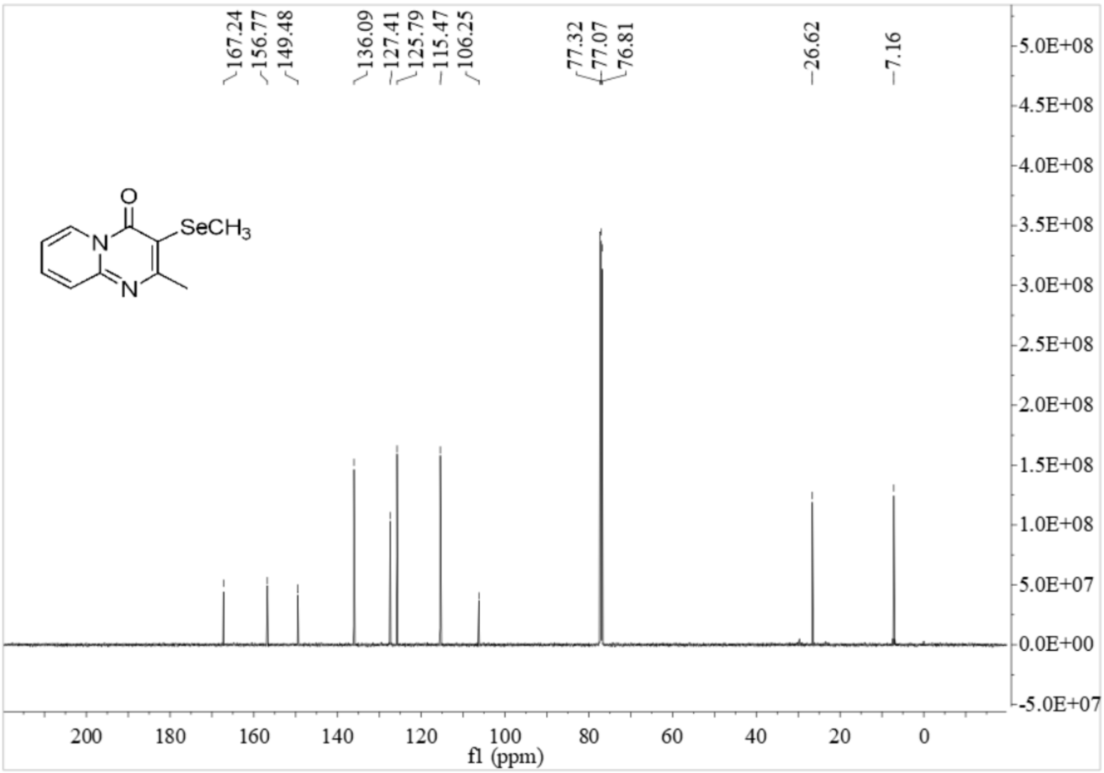

Compound 3ae

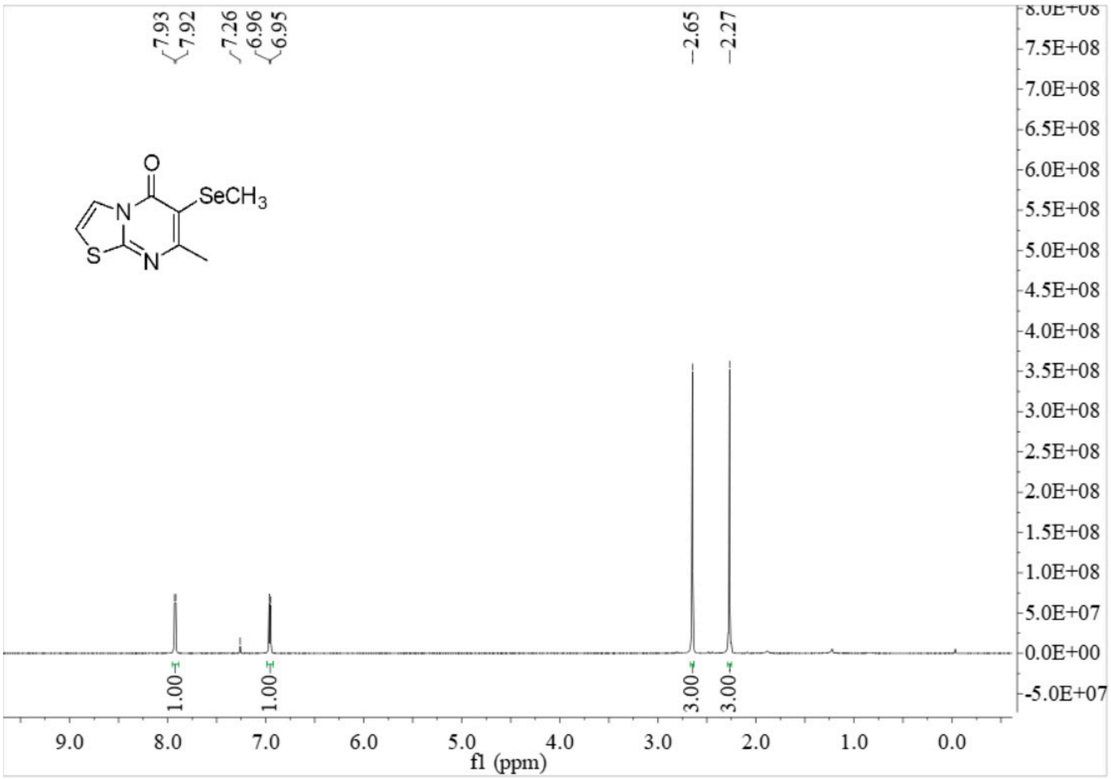

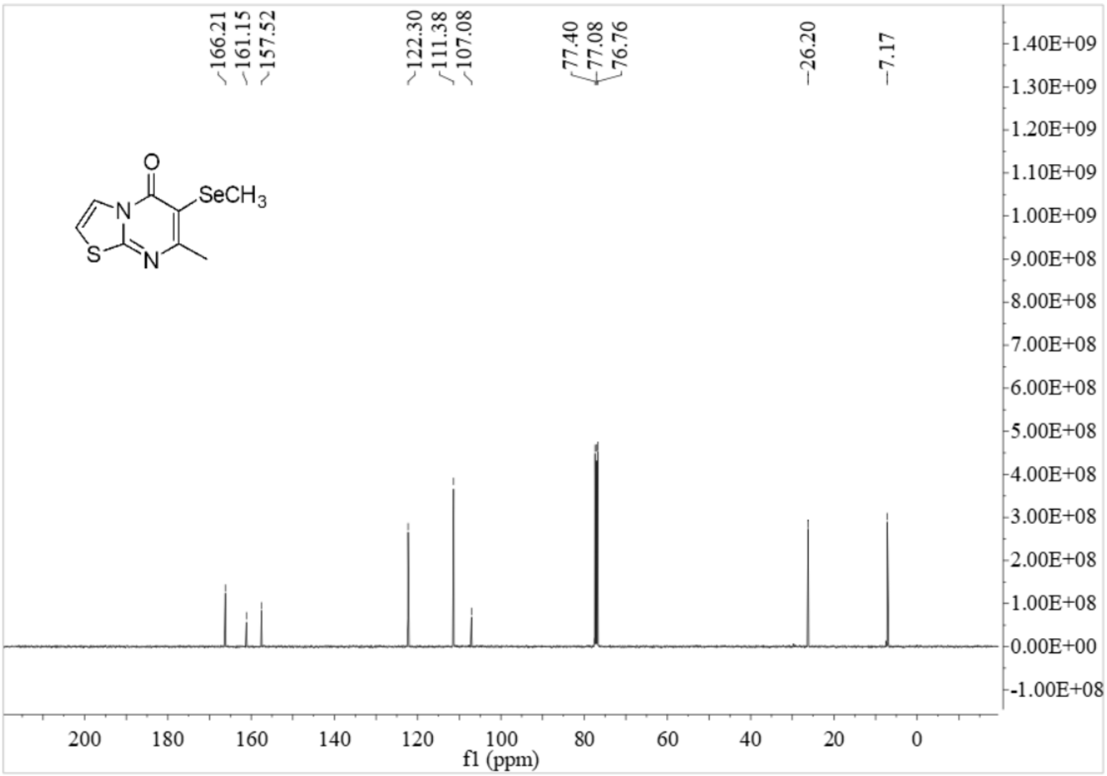

Supplement: Supplementary file 1 [file molecules-28-02206-s001.zip › molecules-2218164-supplementary.pdf]
